# *Yaravirus brasiliense* genomic structure analysis and its possible influence on the metabolism

**DOI:** 10.1590/1678-4685-GMB-2024-0139

**Published:** 2025-02-07

**Authors:** Ana Karoline Nunes-Alves, Jônatas Santos Abrahão, Sávio Torres de Farias

**Affiliations:** 1 Universidade Federal da Paraíba, Departamento de Biologia Molecular, Laboratório de Genética Evolutiva Paulo Leminski, João Pessoa, PB, Brazil.; 2 Network of Researchers on the Chemical Evolution of Life (NoRCEL), Leeds, United Kingdom.; 3 Universidade Federal de Minas Gerais, Instituto de Ciências Biológicas, Departamento de Microbiologia, Laboratório de Vírus, Belo Horizonte, MG, Brazil.

**Keywords:** Auxiliary metabolic genes, viral evolution, citric acid cycle, genetic structure

## Abstract

Here we analyze the *Yaravirus brasiliense*, an amoeba-infecting 80-nm-sized virus with a 45-kbp dsDNA, using structural molecular modeling. Almost all of its 74 genes were previously identified as ORFans. Considering its unprecedented genetic content, we analyzed Yaravirus genome to understand its genetic organization, its proteome, and how it interacts with its host. We reported possible functions for all Yaravirus proteins. Our results suggest the first ever report of a fragment proteome, in which the proteins are separated in modules and joined together at a protein level. Given the structural resemblance between some Yaravirus proteins and proteins related to tricarboxylic acid cycle (TCA), glyoxylate cycle, and the respiratory complexes, our work also allows us to hypothesize that these viral proteins could be modulating cell metabolism by upregulation. The presence of these TCA cycle-related enzymes specifically could be trying to overcome the cycle’s control points, since they are strategic proteins that maintain malate and oxaloacetate levels. Therefore, we propose that Yaravirus proteins are redirecting energy and resources towards viral production, and avoiding TCA cycle control points, “unlocking” the cycle. Altogether, our data helped understand a previously almost completely unknown virus, and a little bit more of the incredible diversity of viruses.

## Introduction

Viruses are a type of mobile genetic elements which often encode proteins that encapsidate their own genomes (Koonin *et al.*, 2021). Most of them carry their information in a single molecule in the viral particle (Michalakis and Blanc, 2020). Usually, viruses are seen as simple causative agents of disease, infecting bacteria, archaea, algae, invertebrate and vertebrate animals, plants, but they show exceptional variation and numerous evolutionary strategies (Bekliz *et al.,* 2016). 

Several mechanisms related to viruses of small genomes and limited number of coding genes have been characterized. For example, their genomes can be monopartite (single nucleic acid molecule), segmented (several molecules in the same capsid) or multipartite (several molecules in different capsids) (Bonnamy *et al.*, 2024). Segmentation is a valuable strategy since it increases the total coding potential of a genome and allows exchange of intact genes between related viruses infecting the same cell (Influenza virus’ case) (Lowen, 2018). 

At the same time, it might seem like a segmented or multipartite genome puts a huge challenge on the virus, such as how to pack several genomes and having their entire separate genetic information needing to be present concomitantly during infection. Viruses have numerous strategies to overcome these problems, such as intercellular trafficking, selective packaging, specialized proteins, sorting mechanism, and modulation of gene expression (McDonald and Patton, 2011; Michalakis and Blanc, 2020; Di Mattia *et al.*, 2022; Bonnamy *et al.*, 2024). 

There are also several mechanisms of gene overlap, which is a strategy that allows the synthesis of more proteins while maintaining the same genome length (Pavesi *et al.*, 1997; Chirico *et al.*, 2010). Those mechanisms are used during viral gene expression and include polyprotein synthesis, frameshift changes, and suppression of stop codons. Also, viruses usually lack introns and their intergenic regions are often short or even non-existent (Sabath *et al.*, 2012). Many theories have been proposed to explain the origins of gene overlap, and the most accepted one is gene novelty, which argues that random mutations could introduce a start site on an existing coding gene, resulting in a new open reading frame, overlapping the original gene (Brandes and Linial, 2016). On the other hand, gene overlap has a great evolutionary price, since two functional proteins overlapping can lead to constrained evolution (Mizokami *et al.*, 1997; Brandes and Linial, 2016). 

These mechanisms reflect the plasticity of viral genetic information, and ensure the synthesis of all necessary viral proteins, including those that might also be related to greater viral independence in relation to its host, by redirecting energy and other resources toward the production of viral particles (Blanc-Mathieu *et al.,* 2021; Belhaouari *et al.,* 2022). There is also the theoretical possibility that sequence similarities between foreign and the cell own peptides could result in the cross-activation of cellular pathways (Anand and Tikoo, 2013). When a virus takes over the cell processes and transforms it into a viral factory, the cell becomes a virocell (Forterre, 2013).

In recent years, there have been several reports of metabolism-related genes in viruses which give them the potential to actively regulate it with their own genes, even though viruses are often seen as having limited metabolic capacities (Blanc-Mathieu *et al.,* 2021; Pant *et al.,* 2021). These genes are related to photosynthesis, light harvesting, tricarboxylic acid (TCA) cycle, pentose phosphate pathway, glycolysis, gluconeogenesis, β-oxidation, protein synthesis, aminoacyl-tRNA synthetases, tRNAs, cytoskeletal structure, nutrient transport, nitrogen cycle, vesicular trafficking, and fermentation (Needham *et al.,* 2019; Moniruzzaman *et al.,* 2020, 2023; Sun *et al.,* 2020; Blanc-Mathieu *et al.,* 2021; Belhaouari *et al.,* 2022). 

Here, we analyze the *Yaravirus brasiliense*, an amoeba-infecting 80-nm-sized virus with a 44,924-bp dsDNA. Although its genome encodes 74 predicted proteins, almost all of its genes were previously predicted as ORFans. ORFans are coding sequences without homologs in public sequence databases, making it harder to know their origin or function. None of the sequences encoding Yaravirus proteins present similarity with known sequences at a nucleotide level and only six had distant similarities at the amino acid level. In its genome are found six types of tRNAs (tRNA-Asn [gtt], tRNA-Cys [gca], tRNA-His [gtg], tRNA-Ile [aat], tRNA-Ser [tga] and tRNA-Ser [gct]) that do not match commonly used codons (Boratto *et al.*, 2020). Since Yaravirus differs so much from other described amoebae viruses and considering that it encodes for a major capsid protein containing a double-jelly-roll domain (MCP-DJR), it has been placed in its own family, *Yaraviridae*, in the existing realm *Varidnaviria* and kingdom *Bamfordvirae* (Boratto *et al.*, 2022).

Proteomic analysis of the viral particle revealed 26 proteins, including the double jelly roll in the major capsid protein (MCP-DJR) as the most abundant protein present in its icosahedral capsid. Its GC content is around 57%, like its host (*Acanthamoeba castellanii*). The distribution and natural hosts of Yaravirus remain unclear, and it seems extremely uncommon in nature. Yaravirus growth in amoeba cells is fastidious. Either way, it is the first virus isolated in *Acanthamoeba* that potentially does not belong to the nucleocytoplasmic large DNA virus (NCLDVs) (Boratto *et al.*, 2020). 

Herein, considering its unprecedented genetic content, we analyze the Yaravirus genome through threading. Using structural molecular modeling, we seek to understand its genetic organization, its proteome, and how it interacts with its host, including how it possibly manipulates the host metabolism and cellular pathways.

## Material and Methods

### Data sources

Genomic information for *Yaravirus brasiliense* is published in the NCBI genome database (Sayers *et al.*, 2022). The genome used is the updated 2023 version (https://www.ncbi.nlm.nih.gov/nuccore/NC_076895.1) (Boratto *et al.*, 2020). 

### Molecular modeling

We conducted *in silico* structural analyses to characterize the Yaravirus proteins and propose their functions. All 74 predicted protein sequences were modeled in I-TASSER (https://zhanggroup.org/I-TASSER/), a threading-based program (Yang and Zhang, 2015; Zheng *et al.*, 2021; Zhou *et al.*, 2022 a ). I-TASSER constructs 3D structural models by assembling fragments taken from target models and aligning them with known proteins in functional databases. Additionally, it generates structures for unaligned regions through ab initio folding using Monte Carlo simulations with replica exchange. 

An important metric used in our results was TM-score, or template modeling score, which is a measure used to evaluate the structural similarity of protein topologies. It is designed to address important issues found in traditional metrics, such as root-mean-square deviation (RMSD), by increasing the sensitivity to global fold similarity rather than local structural variations and incorporating a length-dependent scale to standardize distance errors. It ranges from 0 to 1, 1 being a perfect match between analyzed structures. Usually, scores below 0.3 indicate random structural similarity, while scores higher than 0.5 are considered to have the same fold (Zhang and Skolnick, 2005).

Furthermore, we also performed structural analyses using Phyre2 (http://www.sbg.bio.ic.ac.uk/~phyre2/), which employs sophisticated remote homology detection techniques to construct 3D models, and predict ligand binding sites (Kelley *et al.*, 2015). Phyre2 has two modes: normal and intensive. While the “normal mode” utilizes a single model to encompass as much of the sequence as feasible, the “intensive mode” employs multiple templates as it seeks to generate models for different segments of the sequence. Due to Yaravirus’ lack of homology to known sequences, a single model never fully covered significant parts of its sequences, so we preferred to use the “intensive mode”.

For a deeper understanding of possible protein ligands, we used the algorithm COACH (https://zhanggroup.org/COACH/) (Yang *et al.*, 2013 a , 2013b). COACH employs a consensus methodology integrating substructure comparison for specific binding (TM-site), sequence profile alignment (S-site), and COFACTOR (a method based on structure, sequence, and protein-protein interactions for annotating biological functions of proteins) to provide complementary predictions of binding sites. The confidence level is indicated by the C-score, ranging from 0 to 1, with higher scores denoting more reliable predictions. For protein visualization, we used PyMol (https://pymol.org/2/) and Swiss-Pdb Viewer (https://swissmodel.expasy.org/) (Guex and Peitsch, 1997). 

### Structural alignment and prediction of functional structure

From the results, it was observed that different proteins were related to the same functions. These proteins were aligned with experimentally resolved template proteins available in the Protein Data Bank (https://www.rcsb.org/) (Berman *et al.,* 2000) through the TM-align tool (https://zhanggroup.org/TM-align/) (Zhang and Skolnick, 2005). We utilized sequence alignment to assess the level of identity among these protein sequences. This approach enables the examination of both similarity and identity between sequences, aiding in the identification of conserved regions. Using the protein visualization programs, these complexes were superimposed and aligned, and the template proteins chains were removed. The remaining chains (i.e., Yaravirus proteins) were inserted into the COACH tool for ligand analysis (https://zhanggroup.org/COACH/) (Yang *et al.*, 2013 a , 2013b). We called those “assembled proteins”.

### Protein structure search

For a better comprehension of the 3D structural models generated by I-TASSER and Phyre2, we used FoldSeek (https://search.foldseek.com/search) (van Kempen *et al.*, 2024), which is a search tool that uses a structural alphabet and tertiary amino acid interactions to align the structure of the inserted protein against several protein databases, such as PDB, AlphaFold Proteome, and CATHY50. 

### Prediction of splicing sites

We performed splicing analyses to identify splicing and alternative splicing sites in Yaravirus genome, yet no such sites were discovered. We used DeepGenGrep, a deep learning-base predictor (Liu *et al.*, 2022); GENSCAN, a probabilistic model for identifying splicing sites through complete exon/intron sequences (Burge and Karlin, 1997); SAPFIR, an alternative splicing tool that links alternative splicing sites to protein features, like functional domains, motifs and sites (Zhou *et al.*, 2022 b ); NetGene2, an artificial neural networks based tool that predicts transition regions between introns and exons to find splicing sites (Brunak *et al.*, 1991; Hebsgaard *et al.*, 1996) and, finally, NetAspGene 1.0, which is a tool that uses single local networks to predict splice sites in the genus *Aspergillus*, whose sequences have smaller introns than average (Wang *et al.*, 2009). 

## Results

### Modeling and structural analysis

All 74 Yaravirus predicted proteins were modeled. The generated models exhibited structural resemblance to various proteins deposited in the Protein Data Bank (PDB). Based on our findings, Yaravirus proteins were classified into five categories: i - basal metabolism; ii - DNA metabolism; iii - RNA metabolism; iv - cell death and protein breakdown, and v - signaling. 57% of the functions were above 0.5, while the others were between 0.302 and 0.498. The only exception was GeneID:80539330, with a TM-score of 0.272. For more information, see Table S1. 

**Figure 1 -:**
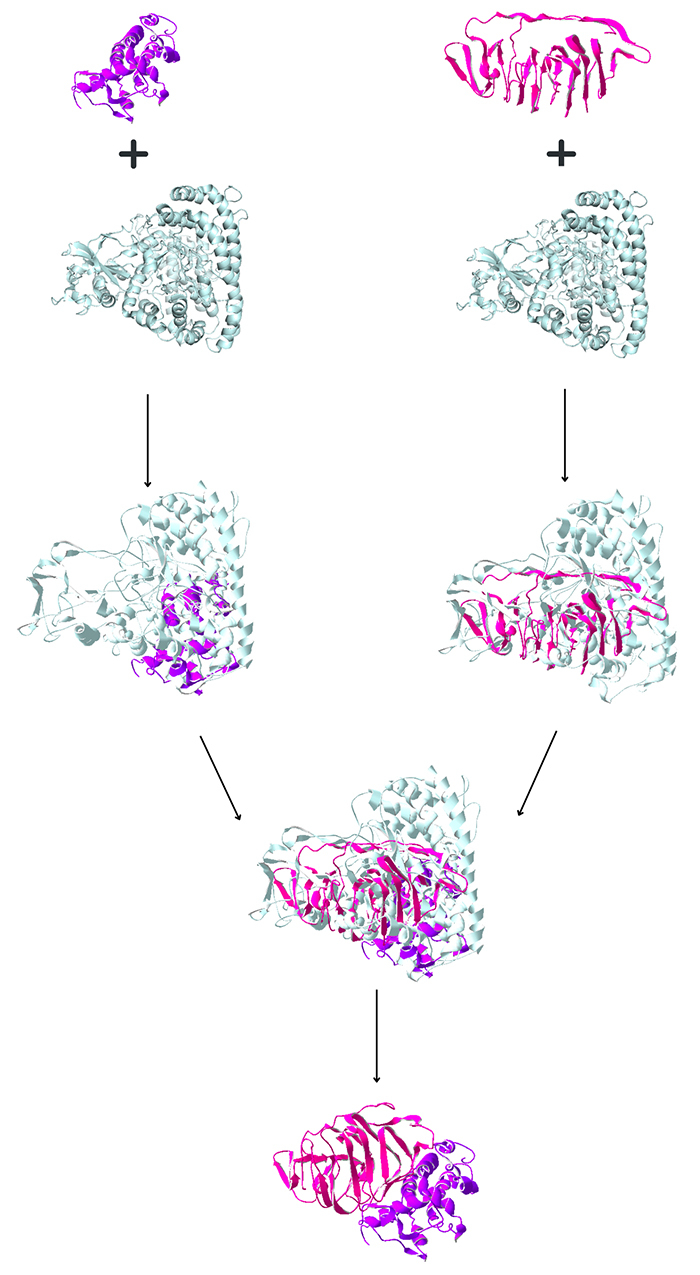
Proposed process to assemble related proteins. Here we show how we assembled two proteins which exhibited structural resemblance to a malate synthase. Purple = GeneID:80539311, pink = GeneID:80539266, light blue = 1Y8B (experimentally resolved protein available in the Protein Data Bank used as a template), purple + light blue = GeneID:80539311 aligned with 1Y8B, pink + light blue = GeneID:80539266 + 1Y8B, light blue + purple + pink = all two proteins aligned with 1Y8B, purple + pink = all two proteins aligned, after removing 1Y8B.

The Yaravirus genome codes for several small proteins. When analyzing structural similarities, we observed that 58 of the encoded proteins had the same function with at least one other protein. It is important to note that most of these proteins are not low complexity fragments, as can be seen in Table S2. From these data, we conducted structural alignments between the Yaravirus proteins and similar proteins from the Protein Data Bank. The results showed that the different proteins that presented similarities with the same function were structurally aligned in different parts at the same proteins deposited in the PDB, suggesting that these small proteins could be dimerizing to reestablish the predicted function (Figure S1, S2and S3). Figure 1 shows how we assembled these proteins, as described in Materials and Methods. We were able to assemble 26 proteins composed of at least two Yaravirus proteins for the same function. These proteins are involved in many biological processes such as central carbon metabolism, DNA metabolism, RNA metabolism, tRNA metabolism, cell wall breakdown, and cell signalization.

**Figure 2 -:**
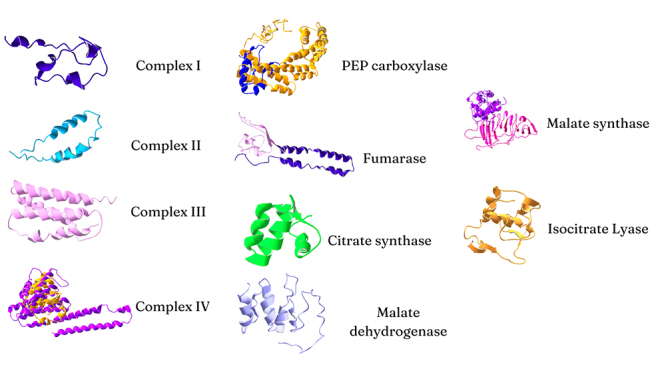
TCA cycle, glyoxylate cycle, and respiratory complexes related proteins.

From structural analysis, we observed that 10 proteins encoded in the Yaravirus genome had similarities with proteins of the central carbon metabolism, such as tricarboxylic acid (TCA) cycle, the glyoxylate cycle, and the mitochondrial respiratory complexes (Figure 2). The location and gene identification of these proteins can be found in Table S3. 

### Ligand analysis

By conducting comparative analyses of sequence alignments and homologous structures, we estimated potential ligands for Yaravirus proteins and assembled proteins, using the COACH tool. Among the results obtained we can highlight the DNA-dependent DNA-polymerase binds to magnesium, which is important for its normal functioning (Yang *et al.*, 2004), and mercury, an inhibitor (Williams *et al.*, 1987). DNA topoisomerase has affinity with ATP and magnesium, as expected (Baird *et al.,* 1999; Sissi and Palumbo, 2009), and also with copper complexes, which have inhibitory action on topoisomerases (Molinaro *et al.*, 2020). DNA ligase binds to nucleic acid, magnesium, manganese and zinc (Yang *et al.*, 1996; Lee *et al.*, 2000; Sriskanda *et al.*, 2000; Taylor *et al.*, 2011). Serine tRNA ligase has affinity to zinc and magnesium, while cobalt inhibits it (Kalogerakos *et al.*, 1979; Bilokapic *et al.*, 2006; Saha and Nandi, 2022). Finally, DNA helicase is inhibited by mercury and binds to calcium and zinc (Bernstein *et al.*, 2003; Chen *et al.*, 2011; Li *et al.*, 2016).

Table S3 provides additional data about the proteins related to the TCA cycle, glyoxylate cycle, and respiratory complexes and their ligands.

## Discussion

### Comparison with **Boratto *et al.* (2020**)

There are three approaches to *in silico* protein prediction: homology modeling, threading (or fold recognition), and *ab initio* (or template free) (Venkatesan *et al.*, 2013). Homology modeling is considered most effective for straightforward targets, which are sequences with abundant homologous information available in public sequence databases. This method heavily depends on a high sequence identity between the target (the protein the user aims to predict) and the template (the experimental structure of a closely related protein) to generate accurate models. Consequently, predicting hard targets, i. e., proteins lacking sufficient homologous information in sequence databases, becomes more challenging (Peng and Xu, 2010). 

Threading, which is the method used in this paper, can identify sequences with similar protein folds that have less sequence similarity, because it considers both sequence and structural similarity. That means, when there is no significant homology found, the threading methods make predictions based on structural information. Finally, *ab initio* constructs proteins from scratch, rather than using available solved structures, relying entirely on biophysical principles to generate models. Harder targets, usually considered those with sequence alignment lower than 25% (such as Yaravirus sequences), are better suited by threading and/or *ab initio* tools.

Boratto *et al*(2020) were able to find functions for 17 out of the 74 Yaravirus predicted proteins using HHpred, Phyre2, and Swiss-Model tools for structural prediction and BLASTp for comparisons. HHpred and Swiss-Model are homology-based tools (Söding *et al.,* 2005; Biasini *et al.*, 2014), which are not considered the best for difficult cases such as Yaravirus (Peng and Xu, 2010). Phyre2, as mentioned, has different available approaches and, to our knowledge, the analysis run by Boratto *et al.* (2020) were run in “normal mode”, which is not the best fit for Yaravirus since it utilizes a single model rather than multiple templates for different segments of the sequence. Perhaps this methodological difficulty is the reason they could only identify functions for 17 out of 74 proteins. 

For the purpose of comparison between Phyre2 and I-TASSER, we remodeled the proteins using Phyre2 in “intensive mode”. I-TASSER generates structural models by assembling fragments taken from target models and aligning them with known proteins from functional databases, comparably to how Phyre2’s “intensive mode” utilizes various templates to create models for distinct segments of the input sequence. To compare our results with their findings, we performed structural similarity analysis in FoldSeek using I-TASSER’s tridimensional models. We were able to confirm their predictions in seven out of those seventeen proteins, as shown in Table 1.

**Table 1 -:** Yaravirus proteins with confirmed functional prediction in comparison to Boratto et al, 2020 [22].

GeneID	Predicted function	Foldseek result (e-value)
GeneID:80539267	Adiponectin	Cerebellin (7.93e-3), adiponectin (6.73e-2)
GeneID:80539268	Cerebellin 1	Adiponectin (1.86e-6), cerebellin (4.36e-6)
GeneID:80539258	Exonuclease	Exonuclease (1.79e-10)
GeneID:80539282	Retinoblastoma-binding protein	Topoisomerase (9.25e+0) and protein-protein binding domains (9.25e+0)
GeneID:80539296	NTPase	NTPase (3.60e-6)
GeneID:80539310	50s ribosomal subunit	60s ribosomal subunits (1.29e-1)
GeneID:80539326	Holliday-junction resolvase	Holliday-junction resolvase (8.36e-6)

GeneID:80539267 and GeneID:80539268, respectively identified originally as an adiponectin and a cerebellin, are very similar structurally, which helps explain why they are both a match for cerebellin and adiponectin. GeneID:80539258 was identified as an exonuclease several times by different approaches. Boratto *et al.* (2020) identified this function using BLASTp and *in silico* prediction, and we were able to confirm it with Phyre2, I-TASSER, and FoldSeek.

GeneID:80539282 was linked to a retinoblastoma-binding protein, which is a type of protein that is related to regulation of transcription and differentiation. Our FoldSeek results linked this protein to a topoisomerase domain, which is an enzyme essential for DNA replication and transcription, and a protein-protein binding domain, therefore confirming its original result. GeneID:80539296, recognized as a NTPase was confirmed as a NTPase by Foldseek. GeneID:80539310 matches several times as 60s and 50s ribosomal subunits. Finally, GeneID:80539326 was confirmed as a Holliday-junction resolvase.

Only four models generated by Phyre2 had more than 0% residues modeled at >90% confidence, and only two had more than 70% confidence (GeneID:80539258 and GeneID:80539297). Therefore, I-TASSER results were better than Phyre2’s, by alignment coverage and confidence parameters. This is probably due to the fact that in highly challenging modeling scenarios, such as when remote homology is uncertain and substantial portions of a sequence cannot be aligned with a known structure, which is precisely Yaravirus’ case, I-TASSER has better results than Phyre2 (Kelley *et al.*, 2015). 

One of the noteworthy exceptions was GeneID:80539297, which had 74% of residues modeled at >90% confidence. It was identified by Phyre2 as a Double-Jelly Roll domain of a Major Capsid Protein (DJR-MCP), confirming the results found by Boratto *et al.* (2020). It is also worth mentioning that this protein was the most abundant one in the proteome analysis (Boratto *et al.*, 2020). Perhaps this isolated result is due to DJR-MCP being a more structurally conserved protein (Krupovic *et al.*, 2022).

**Figure 3 -:**
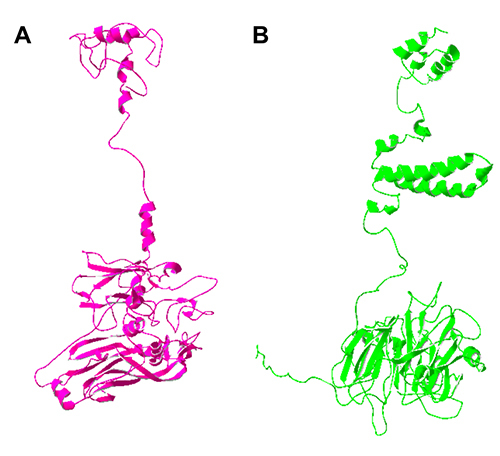
A (pink) is Phyre2’s 3D structural model; B (green) is i-TASSER’s model.

I-TASSER’s model is structurally similar to Phyre2’s model (Figure 3). We inserted both models in FoldSeek for better understanding, and while Phyre2’s seems related to the DJR-MCP, I-TASSER’s is like vacuolar proteins related to fusion, such as the homotypic fusion and vacuole protein sorting (HOPS) complex. In laboratory conditions, there is evidence of Yaravirus entry into the cell by endocytic vesicles, which could explain this result (Boratto *et al.*, 2020), as well as the fact that different viruses also use endocytosis as an entry mechanism, such as Hepatitis C Virus (HCV) and Marburg Virus (Koutsoudakis *et al.*, 2007; Kolesnikova *et al.*, 2009). Another hypothesis to explain these differences is that this protein takes on alternative conformations during viral entry: its capsid also functions as a fusion protein, therefore enabling the entry by fusing with the host’s membrane. 

The other exception was GeneID:80539258, which had 78% of residues modeled at >90% confidence. It was identified by Phyre2 and I-TASSER as an exonuclease, confirming the results found by Boratto *et al.* (2020), as previously mentioned.

### Predicted proteins analysis

As mentioned previously, viruses show numerous genomic strategies to overcome the limited number of coding genes, including multipartite genomes, and, perhaps most prevalently, gene overlap (Brandes and Linial, 2016; Bonnamy *et al.*, 2024). Here we propose that genes which are far apart in the genome are being expressed separately as distinct modules of the same protein and joined together at a peptide level, rather than at mRNA level, which would characterize an alternative splicing event. Even if that was the case, no known splicing or alternative splicing sites were found in Yaravirus genome. Therefore, we hypothesize that this could be a new type of genomic organization. 

As with the overlapping genes, the fragmented proteome allows for more proteins within the small genome in the limited capsid, but it can also lead to constrained evolution, since different functional proteins would be going through distinct evolutionary pressures (Mizokami *et al.*, 1997; Brandes and Linial, 2016). Still, the distinct modules allow greater possibilities of combinations. It is known that protein secondary structures are context-dependent, being the result of local conformational preferences and non-local factors, such as tertiary interactions (Minor and Kim, 1996). Also, viral proteins that change conformation have been described in literature, such as the Ebola VP40 protein, which has three different functional assemblies: dimer, hexamer, and octamer (Bornholdt *et al.*, 2013). Therefore, it is possible that when these modules are alone, they can perform a certain function and behave a certain way, but while they are a part of a greater protein, combined with other chains, they perform a different function and behave differently. 

This mechanism suggests the flexibility of viral genetic information, since this combination can allow for new functions while maintaining the same genome length, increasing the viral protein repertoire. These new functions might be related not only to the necessary viral proteins, which are often the ones necessary for viral replication either directly or indirectly, but also to proteins related to strategies for influencing the host physiology during infection, which contribute to increased viral autonomy (Shi and Lai, 2005; Moniruzzaman *et al.*, 2023).

**Figure 4 -:**
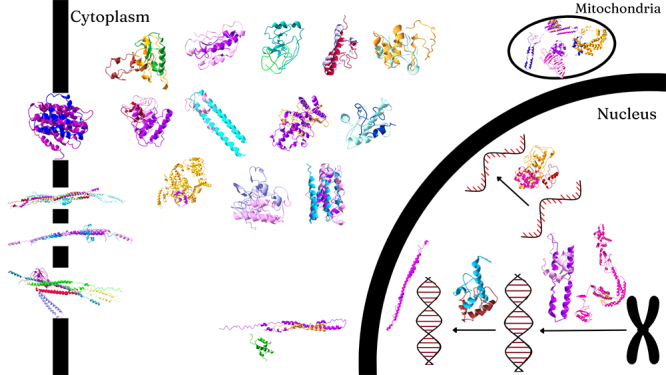
All 26 assembled proteins in the cell environment. Each protein is made of at least two different peptides, represented by the different colors. In the cell membrane, we can find, from top to bottom, an H(+)/K(+)-exchanging ATPase, a histidine kinase, a serine/threonine kinase and a ABC-type xenobiotic transporter. In the cytoplasm, from top to bottom and left to right, we can find a farnesyl diphosphate synthase, a heptaprenyl diphosphate synthase, a triacylglycerol lipase, a beta-n-acetylhexosaminidase, a acyl-CoA dehydrogenase, a glutaryl-CoA dehydrogenase, a nitrite reductase, an endo-1,4-beta-xylanase, a chitinase, a phosphatidylinositol-4,5-bisphosphate 3-kinase, a porphobilinogen synthase, a ribonucleoside-diphosphate synthase and a serine tRNA ligase. In the mitochondria (top right), we can see a fumarase, a malate synthase, a PEP-carboxylase and a complex IV. Finally, in the nucleus, there is an RNA-directed RNA-polymerase, a DNA-directed DNA polymerase, a DNA ligase, a DNA topoisomerase and a DNA helicase. For more information, see Supplementary Material.

An interesting point about this genomic flexibility is that when viruses manipulate the host response, they can rewire host-encoded pathways or even have genes homologous to the host (Rosenwasser *et al.*, 2016). The case reported here and described often in giant viruses (Needham *et al.*, 2019; Moniruzzaman *et al.*, 2020; Sun *et al.*, 2020; Blanc-Mathieu *et al.*, 2021; Belhaouari *et al.*, 2022), is viral auxiliary metabolic genes which are different from the host. We have decided to use the term “auxiliary metabolic gene” (AMG) to describe our findings, since it refers to genes that can exercise control and modulation of host cellular energetic metabolism, even if they are not directly participating in viral replication or are not homologous to the host genes (Rosenwasser *et al.*, 2016; Needham *et al.*, 2019; Sun *et al.*, 2020). Moniruzzaman *et al.* (2023) suggested that these viral genes might be ancient acquisitions from different past hosts, and that these genes might have evolved new functions, not present in their ancestors. Therefore, this is not simply influencing the host physiology or supplementing metabolic demand, but rather a successful strategy for gaining autonomy as the viruses develop new metabolic-related capabilities (Figure 4).

### Metabolism booster and other cellular pathways

From our results, we were able to link Yaravirus proteins to various cellular pathways. Viruses are often seen as having limited metabolic capacities (Blanc-Mathieu *et al.*, 2021; Pant *et al.*, 2021). While there have been several findings in the past few years regarding “cellular like” genes in viruses, such as those involved in protein machinery and the tricarboxylic acid cycle, they have mostly or only been reported in the monophyletic phylum Nucleocytoviricota (Needham *et al.*, 2019; Moniruzzaman *et al.*, 2020; Sun *et al.*, 2020; Blanc-Mathieu *et al.*, 2021; Belhaouari *et al.*, 2022; Moniruzzaman *et al.*, 2023). Both Yaravirus and Nucleocytoviricota share the realm Varidnaviria and the kingdom Bamfordvirae (Boratto *et al.*, 2022), and perhaps this phylogenetic closeness explains the similarities. Rosenwasser *et al.* (2016) have suggested that cells with higher metabolic rates, such as amoebae and algae hosts, are more likely to suffer lytic infection, suggesting an evolutionary pressure that favors dsDNA viruses acquiring and keeping metabolic auxiliary genes. 

Viruses hijacking host processes and resources is not a new concept by any means (Pant *et al.*, 2021). There is evidence of viruses regulating fundamental metabolic processes such as TCA cycle, glycolysis, fatty acid synthesis, amino acid synthesis, and even the increase of nucleotide availability in favor of production of viral particles (Sanchez and Lagunoff, 2015; Thaker *et al.*, 2019; Pant *et al.*, 2021). Besides needing substrates, replication and assembly of viral particles also increase the need of ATP, which is met by these changes in metabolism (Sanchez and Lagunoff, 2015).

Our results showed that several Yaravirus proteins are related to DNA synthesis, repair and recombination, as well as protein synthesis, RNA synthesis and tRNA metabolism (Table S1 and S4). We assembled a ribonucleoside-diphosphate reductase, an enzyme often presents in viral genomes that converts ribonucleotides allowing buildup of deoxynucleotides for viral DNA replication (Turk *et al.*, 1986; Sakowski *et al.*, 2014; Rosenwasser *et al.*, 2016). Viruses usually rely on the host translation machinery, although there are reports of viruses with almost full set of translation-related genes, missing only the complete ribosome (Raoult *et al.*, 2004; Fischer *et al.*, 2010; Philippe *et al.*, 2013; Abrahão *et al.*, 2018). Yaravirus encodes for six types of tRNAs, two of which are serine tRNAs and, interestingly, none of them match the codons most used by neither virus nor host (Boratto *et al.*, 2020). We were able to assemble a serine tRNA ligase , which is an enzyme that attaches amino acids to specific tRNAs. Before the description of giant viruses, this type of enzyme had only been described in cellular organisms. It is not known whether these genes are essential for viral replication, since they could manipulate the host, but it has been hypothesized that these tRNAs could help overcome codon usage differences between virus and host (Duncan *et al.*, 2020). 

We assembled an ABC-type xenobiotic transporter and an H(+)/K(+)-exchanging ATPase , which are large enzymes that use ATP to transport substances across membranes. Potassium channels in viruses and its influence on infection have been described many times (Frohns *et al.*, 2006; Siotto *et al.*, 2014). It has been proposed that viral influence on cell membrane transporters could be related either to depolarization of the cell membrane, which can facilitate a virion’s entry into the cell and avoid multiple infections, or the nutrient intake during infection (Frohns *et al.*, 2006; Greiner *et al.*, 2009; Monier *et al.*, 2017; Greiner *et al.*, 2018; Moniruzzaman *et al.*, 2023). After the infection, it is possible to find Yaravirus particles attached to the outside of the host membrane, therefore it could be possible that this influences its entry (Boratto *et al.*, 2020).

Kinases are enzymes that transfer a phosphate group from ATP to an amino acid residue of a protein. We assembled three proteins related to this function: a histidine kinase, a serine/threonine kinase and a phosphatidylinositol-4,5-bisphosphate-3-kinase . Kinases might have a role in the nuclear takeover during infection, by participating in capsid assembly (Krosky *et al.*, 2003; Jacob *et al.*, 2011), and Yaravirus viral fabric does occupy the nucleus region during infection (Boratto *et al.*, 2020). 

Interestingly, we were able to assemble two enzymes related to cell wall breakdown: a chitinase and an endo-1,4-β-xylanase (Table S2), respectively a chitin-breaking and a cellulose-breaking enzymes. Yaravirus host, *Acanthamoeba castellanii,* is a free-living protozoan, known for its resistant cysts. While many amoebae have chitin in their cyst wall (Campos-Góngora *et al.*, 2004), the *Acanthamoeba* genus has both chitin and cellulose (Linder *et al.*, 2002; Wang *et al.*, 2023). It has been theorized that algae viruses that also have cell wall degrading enzymes use them to penetrate the rigid cell wall (Van Etten *et al.*, 2017). Since Yaravirus is an amoeba virus, we suggest that these enzymes could be avoiding encystment and allowing viral infection to continue. 

**Figure 5 -:**
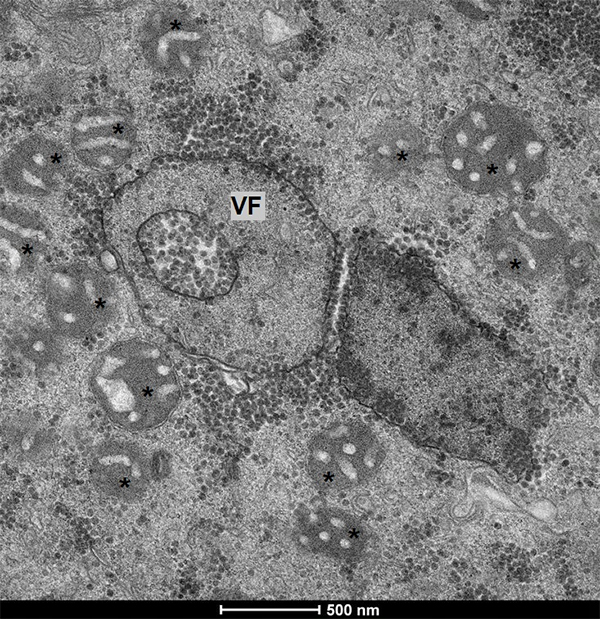
Transmission electron microscopy of an *Acanthamoeba castellanii* cell infected by Yaravirus. This image shows mitochondria (marked by asterisks) surrounding the Yaravirus viral factory (VF). (Scale bar: 500 nm.)

**Figure 6 -:**
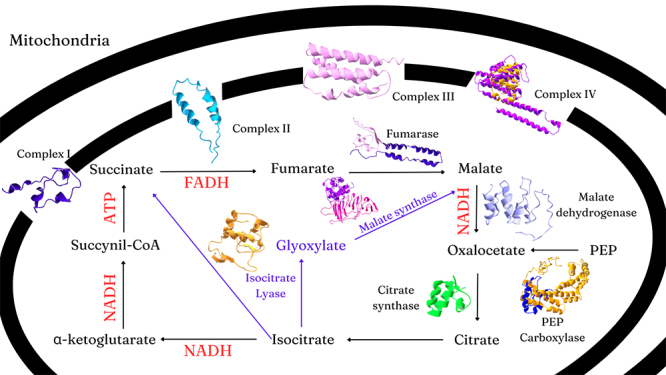
TCA cycle, glyoxylate cycle, and respiratory complexes related proteins. Different colors mean different peptides.

No known viruses code entire metabolic pathways. However, coding key enzymes can raise viral autonomy since the virus relies less on cellular genes. Given the structural resemblance between some Yaravirus proteins and proteins related to the TCA cycle, glyoxylate cycle, and the mitochondrial respiratory complexes , it is possible that these ORFans also take on these functions. Boratto *et al.* (2020) observed in laboratory conditions that Yaravirus attracts mitochondria to the viral factory (Figure 5). In Figure 6, it shows a suggestion of how these proteins could affect the host mitochondria, by manipulation of the mentioned biochemical pathways. 

The tricarboxylic acid cycle (also called Krebs cycle or citric acid cycle) is a series of enzymatic reactions that releases energy through the oxidation of acetyl-CoA. It is linked to the electron transport chain (ETC) at the respiratory complex II, also called succinate dehydrogenase. 

The electron transport chain (ETC) is a series of complexes that transfer electrons via redox reactions with a transfer of protons across the mitochondrial membrane, which drives the synthesis of ATP. Several metabolic pathways take place inside the mitochondria, including the tricarboxylic acid cycle. Yaravirus genome has proteins related to all four respiratory complexes (Figure 2). We hypothesize that these proteins could be upregulating the complexes towards energy production in the form of ATP. Also, succinate dehydrogenase, an enzyme present both in the TCA and the respiratory complexes, is important for avoiding oxidative stress and damage (Dröse, 2013; Moniruzzaman *et al.*, 2020). This is crucial for viral propagation, since it can cause an excess of oxidative damage which can lead to cell death if not mitigated. It is also possible that these proteins are related to apoptosis, since it is known that viruses can manipulate mitochondrial behavior and apoptosis induction (Anand and Tikoo, 2013; Ren *et al.*, 2020; Li *et al.*, 2021; Elesela and Lukacs, 2021; Saxena *et al.*, 2023; Sorouri *et al.*, 2022), but we would need more physiological information to be able to affirm that is the case with Yaravirus.

The TCA cycle provides energy and substrates to other cellular pathways (Goodwin *et al.*, 2015). Its control points are the three exergonic stages (those catalyzed by citrate synthase, isocitrate-dehydrogenase and alpha-ketoglutarate-dehydrogenase); availability of oxaloacetate; and NADH/NAD+ and ATP/ADP ratios. When oxaloacetate levels are low, malate dehydrogenase and citrate synthase are inactivated. When malate levels or oxaloacetate levels are low, the cycle stops. For example, this is due to malate dehydrogenase and citrate synthase being inactivated when oxaloacetate levels are low. On the other hand, when isocitrate levels are high, the cycle also stops. 

We hypothesize that the presence of these TCA cycle-related enzymes could be trying to overcome the control points. As the virus stimulates the cycle to provide energy and substrates for its replication, the control points would eventually reach a limit and be activated. At this point, as shown in Figure 6, Yaravirus malate dehydrogenase and citrate synthase could keep the malate to oxaloacetate to citrate reaction going, and, simultaneously, PEP-carboxylase would also maintain oxaloacetate levels. To keep malate available and to consume isocitrate, the glyoxylate cycle would also be functioning, with the presence of an isocitrate lyase (converts isocitrate to glyoxylate and succinate), a succinate dehydrogenase (converts succinate to fumarate) and a malate synthase (converts glyoxylate to malate). Concurrently, a fumarase converts fumarate to malate, which also maintains malate levels. 

This is not the first report of a virus raising metabolic intermediates levels (Sorouri *et al.*, 2022). Human cytomegalovirus (HCMV) increases TCA substrates levels, while herpes simplex virus type-1 (HSV-1) uses a pyruvate carboxylase to generate intermediates to the TCA cycle (Vastag *et al.*, 2011). Vaccinia virus (VACV) also increases levels of TCA intermediates, especially citrate (Pant *et al.*, 2021). Human adenovirus (HAdV-2) causes changes in pathways that increase the production of TCA intermediates by 1.5 fold, and upregulates certain enzymes present in this cycle (Carinhas *et al.*, 2017; Guissoni *et al.*, 2018; Sánchez-García *et al.*, 2021). 

Our data suggest a new form of genomic organization, where most of the encoded proteins are synthesized as modules and joined together at the protein level. It is also possible to suggest that Yaravirus may be manipulating cellular metabolism, increasing the supply of energy produced to energetically sustain the viral factory. It is interesting to note that other viruses that infect amoebae also present genes related to basal metabolism. This similarity between Yaravirus and other amoeba viruses suggests that there must be a similar selective pressure during amoeba infection that selected these genes in the viruses that infect it.

## Conclusions and final remarks

In the present work, we reported possible functions for all seventy-four of Yaravirus proteins. Altogether, our data help tp understand a previously almost completely unknown virus, and a little bit more of the incredible diversity of viruses yet to be discovered. Our results suggest the first ever report of a fragment proteome, in which the proteins are separated in modules and joined together at a protein level. Our work also allows us to hypothesize that these viral proteins are modulating cell metabolism by upregulating certain enzymes for energy production and viral replication. Our results and conclusions are derived from *in silico* analysis and are limited by the lack of physiological information. Therefore it is important to have further studies with experimental validation, and we suggest they focus on transcriptomics and metabolomics during viral infection to further analyze Yaravirus effect on its host, including which pathways are being promoted or suppressed and how the cell organelles are responding to it.

## Supplementary material

The following online material is available for this article:

Table S1 - Predicted functions for all Yaravirus proteins.

Table S2 - Assembled proteins.

Table S3 - Proteins related to the metabolic pathways.

Table S4 - Structural models for all Yaravirus proteins.

Figure S1 - Assembled proteins related to nucleic acid metabolism and carbon metabolism.

Figure S2 - Assembled proteins related to nitrogen metabolism, mostly.

Figure S3 - Assembled proteins related to general metabolism.
