# Supplementary material for: Yaravirus brasiliense genomic structure analysis and its possible influence on the metabolism
Source: Genet Mol Biol. 2025 Feb 7;48(1):e20240139. doi: 10.1590/1678-4685-GMB-2024-0139 (PMC11803573; doi:10.1590/1678-4685-GMB-2024-0139)
Supplement: Table S1 - [file 1415-4757-GMB-48-1-e20240139-s1.pdf]

Genetics and Molecular Biology
DOI: https://doi.org/10.1590/1678-4685-GMB-2024-0139

Supplementary Material to "*Yaravirus brasiliense* genomic structure analysis and its possible influence on the metabolism"

Table S1 - All proteins. *= present in proteomics.

GENE_ID	Location	Function	Matching proteins
**Amino acid metabolism**			
GeneID:80539260	2033..2236	Amino-acid racemase activity	Proline racemase (PDB code: 1w62A) (RMSD: 3.49) (TM-score: 0.513)
GeneID:80539261	2258..2614	Amino-sugars metabolism	Glucosamine-1-phosphate n-acetyltransferase (PDB code: 2vd4A) (RMSD: 2.69) (TM-score: 0.701)<br>UDP-N-acetylglucosamine diphosphorylase (PDB code: 2vd4A) (RMSD: 2.69) (TM-score: 0.701)
GeneID:80539263*	2913..3215	Alanine, aspartate, and cysteine metabolism	Aspartate 4-decarboxylase (PDB code: 2zy4F) (RMSD: 3.73) (TM-score: 0.506)
GeneID:80539265	3530..3967	Degradative pathway of L-lysine, L-hydroxylysine<br>L-tryptophan metabolism	Glutaryl-CoA dehydrogenase (PDB code: 3ii9C) (RMSD: 4.25) (TM-score: 0.523)
GeneID:80539269	6154..6354	Aspartate and proline metabolism	Aspartate- methyltransferase (PDB code: 1dl5) (RMSD: 3.34) (TM-score: 0.485)<br>4-hydroxyproline epimerase (PDB code: 2azpA) (RMSD: 3.47) (TM-score: 0.506)
GeneID:80539290*	15793..16008	Degradative pathway of L-lysine, L-hydroxylysine<br>L-tryptophan metabolism	Glutaryl-CoA dehydrogenase (PDB code: 2r0nA) (RMSD: 3.40) (TM-score: 0.552)
GeneID:80539293*	16511..16981	Arginine de-imination	Protein-arginine deiminase (PDB code: 1wd9A) (RMSD: 4.19) (TM-score: 0.458)
GeneID:80539298	20741..21025	Shikimate pathway	Chorismate mutase (PDB code: 2gtvX) (RMSD: 3.10) (TM-score:

GENE_ID	Location	Function	Matching proteins
			0.613)
GeneID:80539327*	41680..42048	Serine-type peptidase activity<br>Branched-chain amino acid catabolic process	Hepacivirin (hepatitis C virus NS3 serine proteinase) (PDB code: 1rgqB) (RMSD: 4.96) (TM-score: 0.372)<br>Glutaryl-CoA dehydrogenase (PDB code: 3ii9C) (RMSD: 4.17) (TM-score: 0.401)
**Flavin group metabolism**			
GeneID:80539262*	2626..2913	Riboflavin synthesis	Riboflavin synthase (PDB code: 1kyvE) (RMSD: 3.67) (TM-score: 0.502)
GeneID:80539270	6426..6626	Riboflavin synthesis	Riboflavin synthase (PDB code: 1rvvA) (RMSD: 3.45) (TM-score: 0.494)
**Carbon metabolism**			
GeneID:80539331	1..1025	Monoterpenoids synthesis	Bornyl diphosphate synthase (PDB code: 1n1zA) (RMSD: 2.56) (TM-score: 0.773)
GeneID:80539287	14709..15026	Terpenoids and terpenes synthesis	Farnesyl diphosphate synthase (PDB code: 2azkA) (RMSD: 4.90) (TM-score: 0.488)<br>Heptaprenyl diphosphate synthase (PDB code: 3lmdA) (RMSD: 4.33) (TM-score: 0.493)
GeneID:80539292	16223..16438	Terpenoids and terpenes synthesis<br>Electron transport chain (complex I)	Farnesyl diphosphate synthase (PDB code: 3lomA) (RMSD: 3.24) (TM-score: 0.534)<br>Nitrite reductase (PDB code: 1gu6A) (RMSD: 3.14) (TM-score: 0.548)<br>Complex I (PDB code: 3iamA) (RMSD: 3.42) (TM-score: 0.557)
GeneID:80539278	9611..9808	Electron transport chain (complex II)	Succinate dehydrogenase (PDB code: 1nekC) (RMSD: 2.75)
GeneID:80539319	32828..33178	Electron transport chain (complex III)	Quinol-cytochrome-c reductase (PDB code: 2a06C) (RMSD: 3.97) (TM-score: 0.659)
GeneID:80539294*	17012..17413	Electron transport chain (complex IV)	Cytochrome-C oxidase (PDB code: 2occN) (RMSD: 3.89) (TM-score: 0.510)
GeneID:80539305	25324..26172	Electron transport chain (complex IV)<br>Citric acid cycle flux regulation	Cytochrome-C oxidase (PDB code: 1xmeA) (RMSD: 5.97) (TM-score: 0.454)<br>PEP-carboxylase (PDB code: 1jqnA) (RMSD: 6.15) (TM-score: 0.416)

GENE_ID	Location	Function	Matching proteins
GeneID:80539307	26544..27398	Electron transport chain (complex IV)	Cytochrome-C oxidase (PDB code: 1qleA) (RMSD: 5.69) (TM-score: 0.451)
GeneID:80539264	3248..3478	Tricarboxylic acid cycle	Fumarate hydratase (PDB code: 1vdkA) (RMSD: 3.94) (TM-score: 0.476)
GeneID:80539285	13162..13431	Tricarboxylic acid cycle	Fumarate hydratase (PDB code: 3e04D) (RMSD: 2.32) (TM-score: 0.640)
GeneID:80539291*	16015..16203	Tricarboxylic acid cycle	Citrate synthase (PDB code: 1vgpA) (RMSD: 2.91) (TM-score: 0.558)
GeneID:80539309	28026..28424	Tricarboxylic acid cycle	Malate dehydrogenase (PDB code: 1o0sA) (RMSD: 4.38) (TM-score: 0.557)
GeneID:80539276	8741..8974	Tricarboxylic acid cycle flux regulation	PEP-carboxylase (PDB code: 1jqoA) (RMSD: 3.61) (TM-score: 0.543)<br>Heptaprenyl diphosphate synthase (PDB code: 3lmdA) (RMSD: 3.56) (TM-score: 0.571)<br>Farnesyl diphosphate synthase (PDB code: 2azkA) (RMSD: 3.74) (TM-score: 0.549)
GeneID:80539330*	42456..44924	Tricarboxylic acid cycle flux regulation	PEP-carboxylase (PDB code: 1jqoA) (RMSD: 8.58) (TM-score: 0.272)
GeneID:80539266*	4019..4768	Glyoxylate cycle	Malate synthase (PDB code: 1y8bA) (RMSD: 5.79) (TM-score: 0.434)
GeneID:80539311	28649..29272	Glyoxylate cycle	Malate synthase (PDB code: 2gq3A) (RMSD: 5.70) (TM-score: 0.430)
GeneID:80539316	31020..31409	Glyoxylate cycle	Isocitrate lyase (PDB code: 3i4eA) (RMSD: 4.40) (TM-score: 0.457)
GeneID:80539281	11691..11858	Glycolysis	Phosphoglycerate mutase (PDB code: 1yjxA) (RMSD: 3.29) (TM-score: 0.459)
GeneID:80539259	1779..2036	Reduction of nitrate to ammonia	Nitrite reductase (PDB code: 1gu6A) (RMSD: 2.68) (TM-score: 0.708)
GeneID:80539265	3530..3967	Porphobilinogen synthesis	Porphobilinogen synthase (PDB

GENE_ID	Location	Function	Matching proteins
			code: 1b4eA) (RMSD: 4.36) (TM-score: 0.523)
GeneID:80539295*	17410..17829	Porphobilinogen synthesis	Porphobilinogen synthase (PDB code: 1w1zA) (RMSD: 4.68) (TM-score: 0.444)
GeneID:80539282	11965..12279	Nucleotide sugars metabolism	dTDP-glucose 4,6-dehydratase (PDB code: 1kewA) (RMSD: 3.32) (TM-score: 0.541)
GeneID:80539297*	18954..20744	Methanol metabolism	Methanol dehydrogenase (PDB code: 2d0vA) (RMSD: 5.05) (TM-score: 0.394)
GeneID:80539310	28421..28648	Methane metabolism	Methane hydroxylase (PDB code: 1fziA) (RMSD: 2.10) (TM-score: 0.538)
GeneID:80539329*	42326..42443	Carboxylation of ribulose-1,5-bisphosphate	RuBisCo (PDB code: 1iwaA) (RMSD: 1.90) (TM-score: 0.535)
Lipid metabolism			
GeneID:80539275	8475..8597	Lipase	Triacylglycerol lipase (PDB code: 1qgeD) (RMSD: 3.36) (TM-score: 0.391)
GeneID:80539295*	17410..17829	Lipase	Triacylglycerol lipase (PDB code: 1k8qA) (RMSD: 5.28) (TM-score: 0.453)
GeneID:80539288	15023..15256	Fatty acids metabolism	Acyl-CoA dehydrogenase (PDB code: 1ukwA) (RMSD: 4.26) (TM-score: 0.430)
GeneID:80539309	28026..28424	Fatty acids metabolism	Acyl-CoA dehydrogenase (PDB code: 1ukwA) (RMSD: 4.29) (TM-score: 0.556)
GeneID:80539330*	42456..44924	Fatty-acyl-CoA synthase	Fatty-acyl-CoA synthase (PDB code: 2uv8G) (RMSD: 8.71) (TM-score: 0.302)
**DNA metabolism**			
GeneID:80539258	1054..1782	Exonuclease, DNA binding	Exodeoxyribonuclease (lambda-induced) (PDB number: 1avqA) (RMSD: 2.79) (TM-score: 0.732)
GeneID:80539289*	15274..15756	Exonuclease	Exodeoxyribonuclease III (PDB code: 2o4iA) (RMSD: 4.63) (TM-score: 0.462)
GeneID:80539263*	2913..3215	DNA topoisomerase	DNA topoisomerase (PDB code: 2wl2B) (RMSD: 4.39) (TM-score:

GENE_ID	Location	Function	Matching proteins
			0.486)
GeneID:80539285	13162..13431	DNA topoisomerase	DNA topoisomerase (PDB code: 1sc7A) (RMSD: 2.84) (TM-score: 0.622)
GeneID:80539273	7562..7918	DNA ligase	DNA ligase (PDB code: 1z56A) (RMSD: 1.83) (TM-score: 0.534)
GeneID:80539318	32213..32473	DNA ligase	DNA ligase (PDB code: 1z56A) (RMSD: 3.06) (TM-score: 0.581)
GeneID:80539280	11396..11692	DNA polymerase	DNA-directed DNA-polymerase (PDB code: 1d8yA) (RMSD: 3.24) (TM-score: 0.573)
GeneID:80539296	17852..18907	DNA polymerase	DNA-directed DNA-polymerase (PDB code: 3ikmD) (RMSD: 6.42) (TM-score: 0.418)
GeneID:80539307*	26544..27398	DNA polymerase	DNA-directed DNA-polymerase (PDB code: 3f2bA) (RMSD: 5.83) (TM-score: 0.453)
GeneID:80539315*	30761..30997	DNA polymerase	DNA-directed DNA-polymerase (PDB code: 1s5jA) (RMSD: 3.51) (TM-score: 0.498)
GeneID:80539310	28421..28648	DNA helicase	DNA helicase (PDB code: 2ztdA) (RMSD: 3.71) (TM-score: 0.487)
GeneID:80539326	41010..41588	DNA recombination	Holliday junction resolvase (PDB code: 1hjrC) (RMSD: 2.24) (TM-score: 0.678)
GeneID:80539313	30309..30587	Providing precursors for synthesis and repair of DNA	Ribonucleoside- diphosphate reductase (PDB code: 3ee4A) (RMSD: 3.45) (TM-score: 0.613)
GeneID:80539319	32828..33178	Providing precursors for synthesis and repair of DNA<br>Purine metabolism	Ribonucleoside- diphosphate reductase (PDB code: 1rsrB) (RMSD: 3.59) (TM-score: 0.619)<br>Adenylosuccinate lyase (PDB code: 1yisA) (RMSD: 4.96) (TM-score: 0.416)
GeneID:80539320	33190..33609	Providing precursors for synthesis and repair of DNA	Ribonucleoside- diphosphate reductase (PDB code: 1syyA) (RMSD: 3.83) (TM-score: 0.559)
GeneID:80539271*	6656..6976	Purine metabolism	Adenylosuccinate lyase (PDB code: 1dofA) (RMSD: 3.94) (TM-score: 0.568)
GeneID:80539284*	12564..13049	Histone metabolism	Histone acetyltransferase (PDB: 1z4rA) (RMSD: 2.91) (TM-score: 0.624)
**RNA metabolism (general)**			
GeneID:80539296	17852..18907	RNA polymerase	RNA-directed RNA-polymerase (PDB code: 3evfA) (RMSD: 4.92) (TM-score: 0.407)
GeneID:80539308	27454..27867	RNA polymerase	RNA-directed RNA-polymerase

GENE_ID	Location	Function	Matching proteins
			(PDB code: 2r7oA) (RMSD: 4.67) (TM-score: 0.525)
GeneID:80539315*	30761..30997	RNA polymerase	RNA-directed RNA-polymerase (PDB code: 3mmpG) (RMSD: 3.83) (TM-score: 0.487)
GeneID:80539312	29663..30295	Ribosome-inactivating protein	rRNA N-glycosylase (PDB code: 2g5xA) (RMSD: 5.22) (TM-score: 0.332)
**tRNA metabolism**			
GeneID:80539272	7005..7565	Serine tRNA ligase	Serine tRNA ligase (PDB code: 2zr3B) (RMSD: 3.54) (TM-score: 0.440)
GeneID:80539274*	8034..8339	Serine tRNA ligase	Serine tRNA ligase (PDB code: 3errA) (RMSD: 4.15) (TM-score: 0.508)
GeneID:80539285	13162..13431	Serine tRNA ligase	Serine tRNA ligase (PDB code: 2zr3B) (RMSD: 3.10) (TM-score: 0.644)
GeneID:80539298	20741..21025	Alanine tRNA ligase	Alanine tRNA ligase (PDB code: 2ztgA) (RMSD: 2.62) (TM-score: 0.589)
GeneID:80539314	30613..30756	Valine tRNA ligase	Valine tRNA ligase (PDB code: 1gaxA) (RMSD: 3.09) (TM-score: 0.553)
GeneID:80539283	12276..12545	tRNA nucleotidyltransferase	tRNA nucleotidyltransferase (PDB code: 3dd6A) (RMSD: 3.09) (TM-score: 0.557)
GeneID:80539324*	38002..38211	tRNA-guanosine(34) preQ1 transglycosylase	tRNA-guanosine(34) preQ1 transglycosylase (PDB code: 1q2rA) (RMSD: 3.55) (TM-score: 0.515)
GeneID:80539286	13459..13543	tRNA-Ser	-
GeneID:80539252	13545..13618	tRNA-Cys	-
GeneID:80539253	13625..13696	tRNA-Asn	-
GeneID:80539254	13701..13772	tRNA-His	-
GeneID:80539255	13786..13870	tRNA-Ser	-
GeneID:80539256	13874..13947	tRNA-Ile	-
**Cell death**			
GeneID:80539306*	26209..26559	Antimicrobial action by oxidation	Lactoperoxidase (PDB code: 1qgjA) (PDB code: 3.44) (TM-score: 0.518)
**Protein breakdown**			
GeneID:80539279*	9819..10643	Cell wall breakdown	Endo-1,4-beta- xylanase (PDB

GENE_ID	Location	Function	Matching proteins
			code: 2w5fB) (RMSD: 6.22) (TM-score: 0.417)
GeneID:80539280	11396..11692	Cell wall breakdown	Chitinase (PDB code: 2dkvA) (RMSD: 3.94) (TM-score: 0.569)
GeneID:80539328*	42072..42265	Cell wall breakdown	Chitinase (PDB code: 1wvuB) (RMSD: 4.08) (TM-score: 0.468)
GeneID:80539323	37373..37987	Catalase<br>Cell wall breakdown	Cellulase (PDB code: 1ut9A) (RMSD: 4.99) (TM-score: 0.451)<br>Endo-1,4-beta- xylanase (PDB code: 1h12A) (RMSD: 5.37) (TM-score: 0.468)
GeneID:80539313	30309..30587	Catalase<br>Hydrolysis of hexosamine	Catalase (PDB code: 2v8tA) (RMSD: 3.46) (TM-score: 0.609)<br>Beta-N- acetylhexosaminidase (PDB code: 2j62A) (RMSD: 3.68) (TM-score: 0.549)
GeneID:80539282	11965..12279	Hydrolysis of hexosamine	Beta-N- acetylhexosaminidase (PDB code: 2epoB) (RMSD: 3.78) (TM-score: 0.511)
GeneID:80539300	21325..21561	Hydrolysis of xylans and xylobiose	Xylan-1,4-beta- xylosidase (PDB code: 1uhvB) (RMSD: 2.84) (TM-score: 0.564)
GeneID:80539304	24816..25310	Hydrolysis of glucose residues	Glucan-1,4-alpha-glucosidase (PDB code: 2vn7A) (RMSD: 3.64) (TM-score: 0.584)
GeneID:80539268*	5525..6142	Glycopeptide breakdown	Endo-alpha-N-acetilgalactosaminidase (PDB code: 2zxqA) (RMSD: 4.28) (TM-score: 0.474)
GeneID:80539296	17852..18907	Protein breakdown	Flavivirin (NS2B-3 protease) (PDB code: 2pxaB) (RMSD: 5.09) (TM-score: 0.408)
GeneID:80539310	28421..28648	Protease	Protease endopeptidase complex (PDB code: 1j2qH) (RMSD: 3.80) (TM-score: 0.470)
GeneID:80539312	29663..30295	Ubiquitination	Ubiquitin-protein ligase (PDB code: 1fbvA) (RMSD: 4.47) (TM-score: 0.525)
GeneID:80539269	6154..6354	Ubiquitination	Ubiquitin-protein ligase (PDB code: 2z5dA) (RMSD: 2.98) (TM-score: 0.491)
GeneID:80539301	21855..22424	Hyaluronate lyase	Hyaluronate lyase (PDB code: 1i8qA) (RMSD: 4.23) (TM-score: 0.546)
GeneID:80539303*	23890..24771	Pectate lyase	Pectate lyase (PDB code: 1ru4A) (RMSD: 3.50) (TM-score: 0.643)
**Signalization**			
GeneID:80539297	18954..20744	Endocytosis<br>Exocytosis<br>Vacuolar protein	HOPS core complex (PDB code: 8DIT) (RMSD: 2.43) (TM-score: 0.834)

GENE_ID	Location	Function	Matching proteins
GeneID:80539267*	4793..5494	Membrane fusion<br>Virion penetration<br>Receptor binding	Haemagglutinin- esterase fusion glycoprotein (PDB code: 1flcA) (RMSD: 4.88) (TM-score: 0.496)
GeneID:80539322	36810..37268	ABC-type xenobiotic transporter<br>Phosphorylation of hexoses	ABC-type xenobiotic transporter (PDB code: 3g61A) (RMSD: 3.93) (TM-score: 0.582)<br>Hexokinase (PDB code: 1bdgA) (RMSD: 4.51) (TM-score: 0.516)
GeneID:80539259	1779..2036	ABC-type xenobiotic transporter	ABC-type xenobiotic transporter (PDB code: 3g61A) (RMSD: 2.47) (TM-score: 0.733)
GeneID:80539302*	22421..23833	ABC-type xenobiotic transporter	ABC-type xenobiotic transporter (PDB code: 3g61A) (RMSD: 6.51) (TM-score: 0.309)
GeneID:80539272	7005..7565	Histidine kinase<br>ABC-type xenobiotic transporter	Histidine kinase (PDB code: 3i9wA) (RMSD: 4.45) (TM-score: 0.529)<br>ABC-type xenobiotic transporter (PDB code: 3g61A) (RMSD: 3.35) (TM-score: 0.501)
GeneID:80539257	13957..14670	ABC-type xenobiotic transporter<br>Histidine kinase	ABC-type xenobiotic transporter (PDB code: 3g61A) (RMSD: 4.41) (TM-score: 0.466)<br>Histidine kinase (PDB code: 3i9wA) (RMSD: 4.56) (TM-score: 0.440)
GeneID:80539317	31438..32247	Histidine kinase<br>H(+)/K(+)-ATPase<br>ABC-type xenobiotic transporter	Histidine kinase (PDB code: 3i9wA) (RMSD: 4.52) (TM-score: 0.399)<br>H(+)-transporting ATPase (PDB code: 1u7lA) (RMSD: 4.87) (TM-score: 0.382)<br>ABC-type xenobiotic transporter (PDB code: 3g61A) (RMSD: 3.84) (TM-score: 0.375)
GeneID:80539260	2033..2236	Histidine kinase	Histidine kinase (PDB code: 3by9B) (RMSD: 3.47) (TM-score: 0.522)
GeneID:80539278	9611..9808	Histidine kinase<br>Control of cyclic second messengers' levels	Histidine kinase (PDB code: 1b3qA) (RMSD: 2.88) (TM-score: 0.569)<br>Cyclic AMP phosphodiesterase (PDB code: 3ecnB) (RMSD: 3.06) (TM-score: 0.597)
GeneID:80539285	13162..13431	Histidine kinase	Histidine kinase (PDB code: 3i9wA) (RMSD: 3.22) (TM-score: 0.624)
GeneID:80539318	32213..32473	Serine/threonine protein kinase<br>Histidine kinase<br>ABC-type xenobiotic transporter	Serine/threonine protein kinase (PDB code: 1wt6B) (RMSD: 2.87) (TM-score: 0.527)<br>Histidine kinase (PDB code: 3i9wA) (RMSD: 3.46) (TM-score: 0.592)<br>ABC-type xenobiotic transporter (PDB code: 3g61A) (RMSD: 3.44) (TM-score: 0.533)

GENE_ID	Location	Function	Matching proteins
GeneID:80539299	21126..21299	Serine/threonine protein kinase<br>Histidine kinase	Serine/threonine protein kinase (PDB code: 1wt6B) (RMSD: 2.04) (TM-score: 0.772)<br>Histidine kinase (PDB code: 3i9wA) (RMSD: 2.32) (TM-score: 0.788)
GeneID:80539273	7562..7918	Serine/threonine protein kinase<br>ABC-type xenobiotic transporter	Serine/threonine protein kinase (PDB code: 1wt6B) (RMSD: 1.47) (TM-score: 0.514)<br>ABC-type xenobiotic transporter (PDB code: 3g61A) (RMSD: 3.80) (TM-score: 0.524)
GeneID:80539277	9290..9469	Serine/threonine protein kinase	Serine/threonine protein kinase (PDB code: 1tkiB) (RMSD: 2.87) (TM-score: 0.496)
GeneID:80539308	27454..27867	H(+)/K(+)-ATPase	H(+)/K(+)-ATPase (PDB code: 3ixzA) (RMSD: 4.61) (TM-score: 0.550)
GeneID:80539312	29663..30295	H(+)/K(+)-ATPase<br>Na(+)/K(+)-ATPase	H(+)/K(+)-ATPase (PDB code: 3ixzA) (RMSD: 5.39) (TM-score: 0.488)<br>Na(+)/K(+)-ATPase (PDB code: 3b8eA) (RMSD: 5.28) (TM-score: 0.487)
GeneID:80539316	31020..31409	Phosphorylation	Phosphatidylinositol-4,5-bisphosphate 3-kinase (PDB code: 2x38A) (RMSD: 4.10) (TM-score: 0.471)
GeneID:80539321	33652..36777	Phosphorylation	Phosphatidylinositol-4,5-bisphosphate 3-kinase (PDB code: 2rd0A) (RMSD: 4.44) (TM-score: 0.390)<br>Phosphatidylinositol 3-kinase (PDB code: 3ihyC) (RMSD: 4.39) (TM-score: 0.393)
GeneID:80539329*	42326..42443	Pyridoxal kinase	Pyridoxal kinase (PDB code: 1vi9A) (RMSD: 2.25) (TM-score: 0.529)
