# Supplementary material for: Yaravirus brasiliense genomic structure analysis and its possible influence on the metabolism
Source: Genet Mol Biol. 2025 Feb 7;48(1):e20240139. doi: 10.1590/1678-4685-GMB-2024-0139 (PMC11803573; doi:10.1590/1678-4685-GMB-2024-0139)
Supplement: Table S2 - [file 1415-4757-GMB-48-1-e20240139-s2.pdf]

Genetics and Molecular Biology
DOI: https://doi.org/10.1590/1678-4685-GMB-2024-0139

Supplementary Material to "*Yaravirus brasiliense* genomic structure analysis and its possible influence on the metabolism"

Table S2 - Assembled proteins; **.

Function	GENE_ID	Location	COACH (c-score)	RMSD
Malate synthase	GeneID:80539266 + GeneID:80539311	4019..4768 28649..29272	Thiosulfate (0.02)	5.81 5.70
PEP-carboxylase	GeneID:80539276 + GeneID:80539305	8741..8974 25324..26172	Oxygen molecule (0.23); Calcium ion (0.22); Manganese ion (0.16); Glutaric acid (0.04); Magnesium ion (0.02)	3.37 6.25
Fumarase	GeneID:80539264 + GeneID:80539285	3248..3478 13162..13431	Magnesium ion (0.37); ATP (0.10); L-malate (0.05); Manganese ion (0.02); Iron ion (0.02); Aspartic acid (0.01)	2.91 4.40
Complex IV	GeneID:80539294 + GeneID:80539307	17012..17413 26544..27398	Carbon monoxide (0.05)	4.42 5.69
RNA-directed RNA-polymerase	GeneID:80539296 + GeneID:80539308 + GeneID:80539315	17852..18907 27454..27867 30761..30997	ATP (0.04); Calcium ion (0.04); Zinc ion (0.04); Manganese ion (0.04); Magnesium ion (0.03); Nicotinamide (0.01)	6.42 5.16 4.98
DNA-directed DNA polymerase	GeneID:80539280 + GeneID:80539315	11396..11692 30761..30997	Mercury ion (0.07); Magnesium ion (0.02)	3.24 3.63
DNA topoisomerase	GeneID:80539263 + GeneID:80539285	2913..3215 13162..13431	ATP + Magnesium ion (0.35); Copper ion + Magnesium ion (0.21); NAD (0.16); ATP (0.07); Magnesium ion (0.02)	2.84 4.42
DNA ligase	GeneID:80539273 + GeneID:80539318	7562..7918 32213..32473	Calcium ion (0.14); Zinc ion (0.13); Manganese ion (0.12); Nucleic acid (0.05); Magnesium ion (0.02)	1.83 3.06
DNA helicase	GeneID:80539310 + GeneID:80539325**	28421..28648 38367..40940	Mercury ion (0.14); Zinc ion (0.03); Calcium ion (0.03)	3.71 5.70
Serine tRNA ligase	GeneID:80539272 + GeneID:80539274 + GeneID:80539285	7005..7565 8034..8339 13162..13431	Zinc ion (0.16); Cobalt ion (0.03) Magnesium ion (0.02)	2.78 3.46 4.31
Serine/threonine protein kinase	GeneID:80539273 + GeneID:80539277 + GeneID:80539299 + GeneID:80539318	7562..7918 9290..9469 21126..21299 32213..32473	Manganese ion (0.08); Peptide (0.05); Palmitoyl-linoleoyl-phosphatidylcholine (0.04); Calcium (0.02)	2.07 2.26 2.07 3.02
Histidine kinase	GeneID:80539260 + GeneID:80539272 +	2033..2236 7005..7565	Magnesium ion (0.04); Calcium ion (0.03);	3.76 4.45

Function	GENE_ID	Location	COACH (c-score)	RMSD
	GeneID:80539273 + GeneID:80539278 + GeneID:80539285 + GeneID:80539257 + GeneID:80539299 + GeneID:80539317 + GeneID:80539318	7562..7918 9611..9808 13162..13431 13957..14670 21126..21299 31438..32247 32213..32473	Rubidium ion (0.03); Imidazol (0.03)	2.51 2.84 3.22 4.56 2.32 5.08 3.48
Phosphatidylinositol -4,5-bisphosphate 3-kinase	GeneID:80539316 + GeneID:80539321	31020..31409 42326..42443	Retinol (0.02)	5.05 4.10
Porphobilinogen synthase	GeneID:80539265 + GeneID:80539295	3530..3967 17410..17829	Fructose (0.03); Magnesium ion (0.02); Zinc ion (0.02)	4.36 4.98
Triacylglycerol lipase	GeneID:80539275 + GeneID:80539295	8475..8597 17410..17829	Iodine ion (0.05); Magnesium ion (0.04)	3.36 5.38
Heptaprenyl diphosphate synthase	GeneID:80539276 + GeneID:80539287	8741..8974 14709..15026	Diphosphate (0.03)	3.56 4.24
Beta-n-acetylhexosaminidase	GeneID:80539282 + GeneID:80539313	11965..12279 30309..30587	Zinc ion (0.07); Calcium ionophore (0.07)	3.27 4.00
ABC-type xenobiotic transporter	GeneID:80539259 + GeneID:80539272 + GeneID:80539273 + GeneID:80539257 + GeneID:80539302 + GeneID:80539318 + GeneID:80539322	1779..2036 7005..7565 7562..7918 13957..14670 22421..23833 32213..32473 36810..37268	Phosphatidylglycerol (0.10); Manganese ion (0.10); Magnesium ion (0.03); Phosphatidylcoline (0.03); Histidine (0.03); Maltoside (0.03); Chloresterol (0.02); Zinc ion (0.02)	2.47 3.35 3.80 4.68 6.37 3.44 3.93
Acyl-CoA dehydrogenase	GeneID:80539288 + GeneID:80539309	15023..15256 28026..28424	Glycerol (0.03)	4.71 4.77
Farnesyl diphosphate synthase	GeneID:80539276 + GeneID:80539287 + GeneID:80539292	8741..8974 14709..15026 16223..16438	Copper ion (0.05); Zinc ion (0.04)	4.10 4.30 3.17
Ribonucleoside-diphosphate reductase	GeneID:80539313 + GeneID:80539319 + GeneID:80539320	30309..30587 32828..33178 33190..33609	Hydrogen peroxide + Zinc ion (0.18) Magnesium ion (0.04); Iron ion (0.04); Zinc ion (0.04); Manganese ion (0.04)	3.64 3.45 3.07
Nitrite reductase	GeneID:80539259 + GeneID:80539292	1779..2036 16223..16438	Magnesium ion (0.08); Sulfite ion (0.05); Cobalt ion (0.05); Calcium ion (0.03); Cholesterol (0.03); Heme group (0.03); Manganese ion (0.03)	2.68 3.14
Endo-1,4-beta- xylanase	GeneID:80539279 + GeneID:80539323	9819..10643 37373..37987	Copper ion (0.07)	6.52 5.49
Chitinase	GeneID:80539280 + GeneID:80539328	11396..11692 42072..42265	Calcium ion (0.02); Zinc ion (0.02)	3.94 4.27
Glutaryl-CoA dehydrogenase	GeneID:80539265 + GeneID:80539290 + GeneID:80539327	3530..3967 15793..16008 41680..42048	FAD (0.04)	4.25 3.40 4.17

Function	GENE_ID	Location	COACH (c-score)	RMSD
H(+)/K(+)-exchanging ATPase	GeneID:80539308 + GeneID:80539312	27454..27867 29663..30295	Phosphatidylcholine (0.05); Phosphatidylethanolamine (0.03); AMP-PNP (0.02)	4.26 5.71
