# Supplementary material for: Yaravirus brasiliense genomic structure analysis and its possible influence on the metabolism
Source: Genet Mol Biol. 2025 Feb 7;48(1):e20240139. doi: 10.1590/1678-4685-GMB-2024-0139 (PMC11803573; doi:10.1590/1678-4685-GMB-2024-0139)
Supplement: Table S3 - [file 1415-4757-GMB-48-1-e20240139-s3.pdf]

Genetics and Molecular Biology
DOI: https://doi.org/10.1590/1678-4685-GMB-2024-0139

Supplementary Material to "*Yaravirus brasiliense* genomic structure analysis and its possible influence on the metabolism"

Table S3 - All fifteen TCA cycle, glyoxylate cycle, and respiratory complexes proteins.

Protein	Gene_ID	Location	C-score	RMSD
Citrate synthase	GeneID:80539291*	16015..16203	Cholesterol (0.05)	2.91
Malate dehydrogenase	GeneID:80539309	28026..28424	Phosphate ion (0.07); Zinc ion (0.03); Copper ion (0.03)	4.38
PEP-carboxylase	GeneID:80539276 + GeneID:80539305	8741..8974 25324..26172	Oxygen molecule (0.23); Calcium ion (0.22); Manganese ion (0.16); Glutaric acid (0.04); Magnesium ion (0.02)	3.37 6.25
Fumarase	GeneID:80539264 + GeneID:80539285	3248..3478 13162..13431	Magnesium ion (0.37); ATP (0.10); L-malate (0.05); Manganese ion (0.02); Iron ion (0.02); Aspartic acid (0.01)	2.91 4.40
Complex I	GeneID:80539292	16223..16438	Magnesium ion (0.06); Bedaquiline (0.04); Calcium ion + Iron ion (0.18)	3.42
Complex II	GeneID:80539278	9611..9808	Heme group (0.20)	2.75
Complex III	GeneID:80539319	32828..33178	Calcium ion (0.03)	3.97
Complex IV	GeneID:80539294 + GeneID:80539307	17012..17413 26544..27398	Carbon monoxide (0.05)	4.42 5.69
Malate synthase	GeneID:80539266 + GeneID:80539311	4019..4768 28649..29272	Thiosulfate (0.02)	5.81 5.70
Isocitrate lyase	GeneID:80539316	31020..31409	Iron/Sulfur cluster (0.04)	4.40
