# Supplementary material for: Yaravirus brasiliense genomic structure analysis and its possible influence on the metabolism
Source: Genet Mol Biol. 2025 Feb 7;48(1):e20240139. doi: 10.1590/1678-4685-GMB-2024-0139 (PMC11803573; doi:10.1590/1678-4685-GMB-2024-0139)
Supplement: Table S4 - [file 1415-4757-GMB-48-1-e20240139-s4.pdf]

Genetics and Molecular Biology
DOI: https://doi.org/10.1590/1678-4685-GMB-2024-0139

Supplementary Material to "*Yaravirus brasiliense* genomic structure analysis and its possible influence on the metabolism"

Table S4 - All seventy four Yaravirus' proteins, modeled in I-TASSER. They are shown in the genome's order.

GENE_ID	3d structure
GeneID:80539331	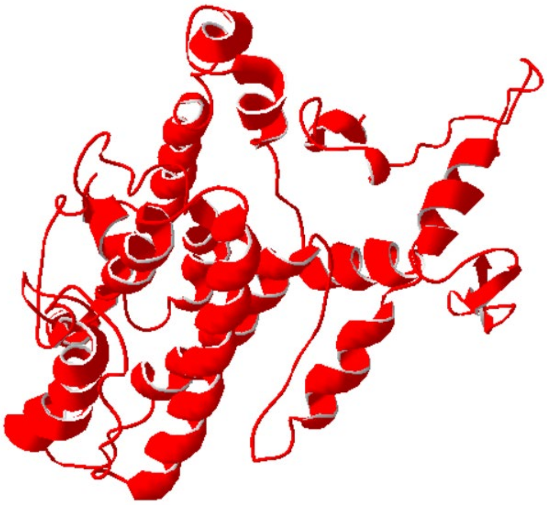
GeneID:80539258	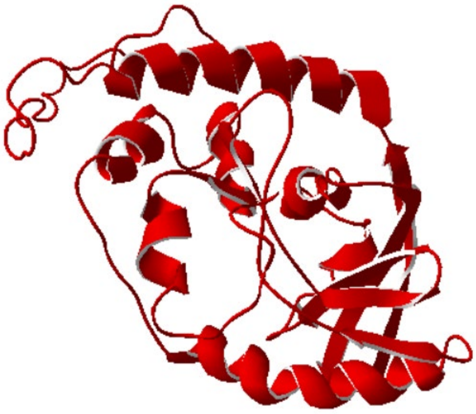
GeneID:80539259	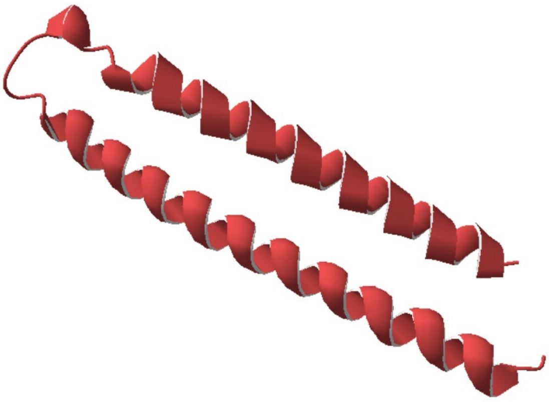

GENE_ID	3d structure
GeneID:80539260	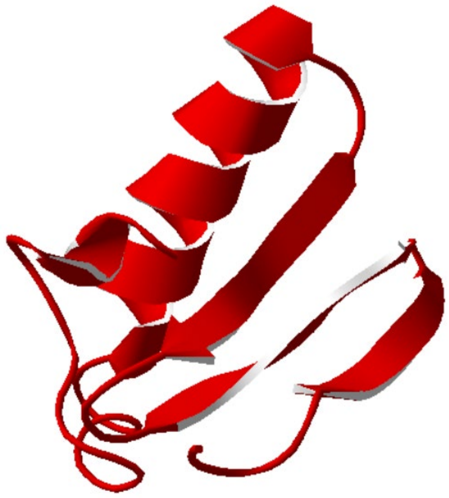
GeneID:80539261	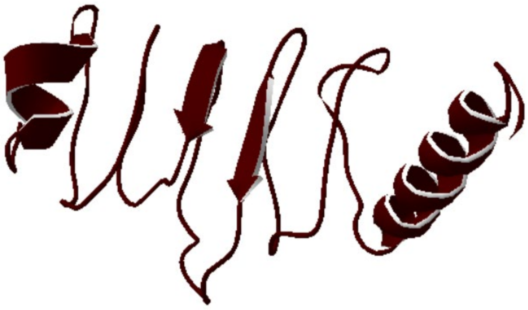
GeneID:80539262	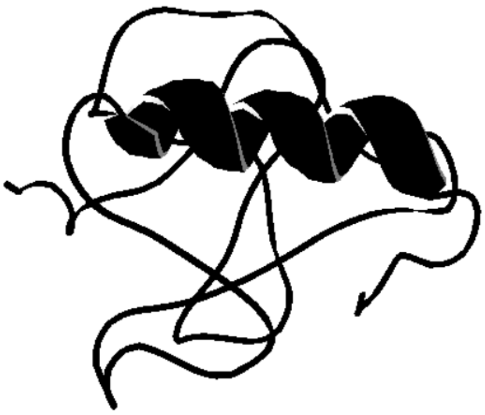

GENE_ID	3d structure
GeneID:80539263	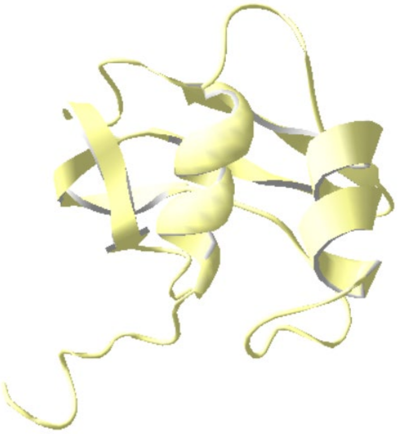
GeneID:80539264	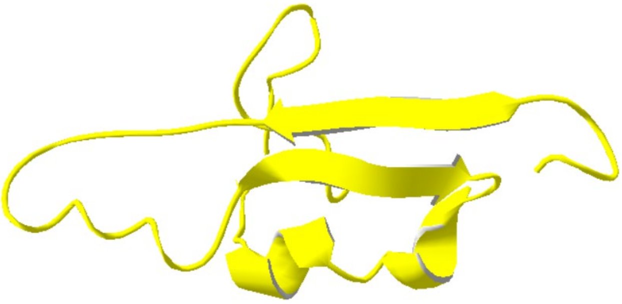
GeneID:80539265	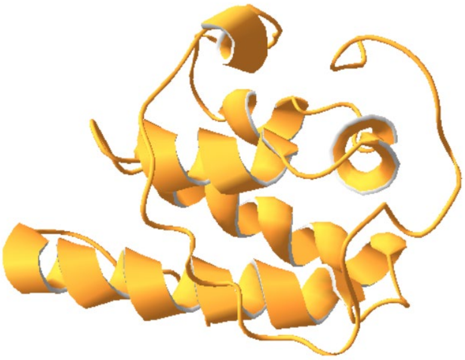
GeneID:80539266	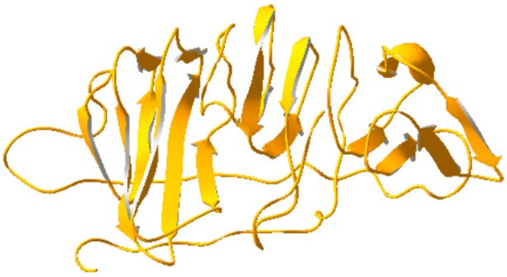

GENE_ID	3d structure
GeneID:80539267	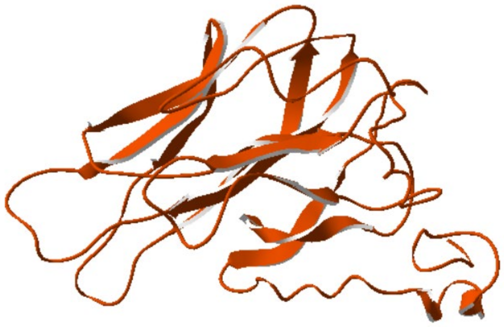
GeneID:80539268	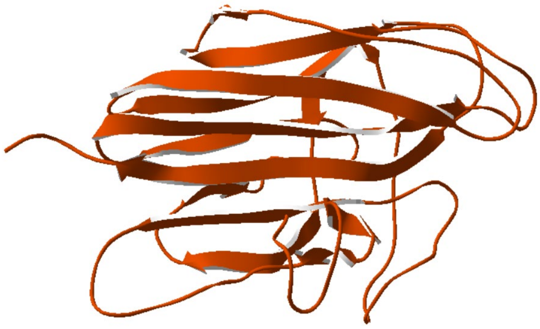
GeneID:80539269	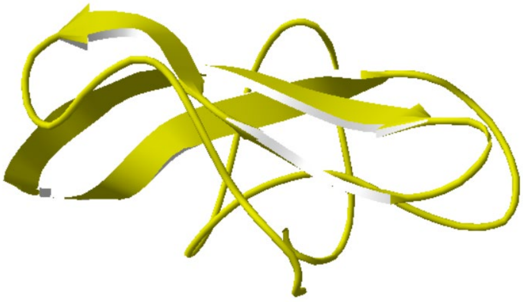
GeneID:80539270	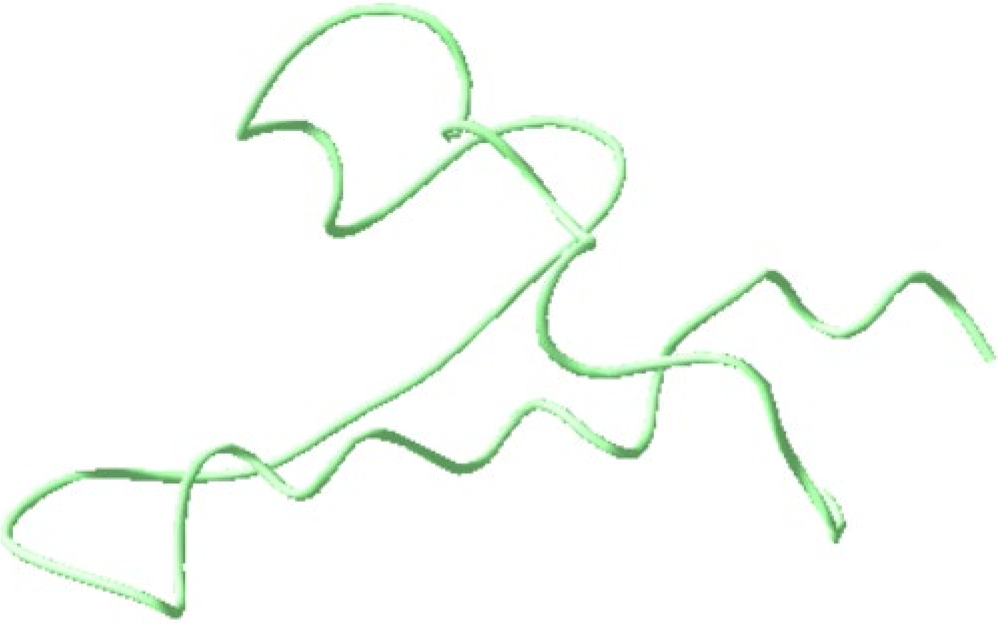

GENE_ID	3d structure
GeneID:80539271	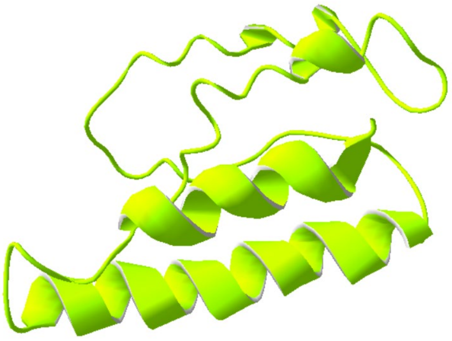
GeneID:80539272	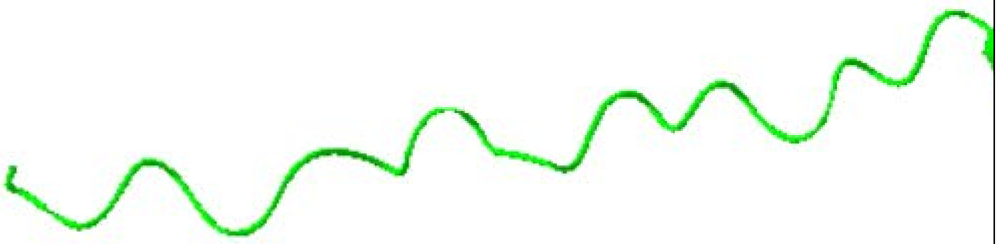
GeneID:80539273	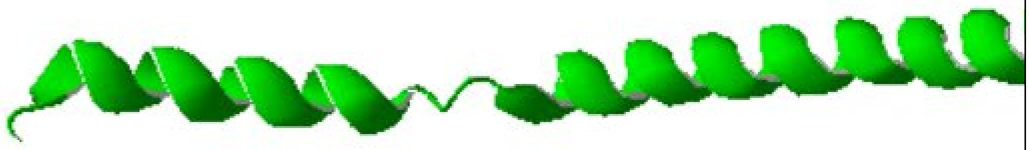

GENE_ID	3d structure
GeneID:80539274	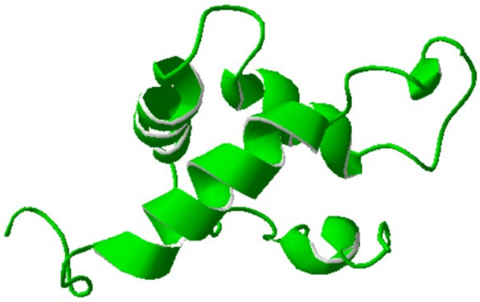
GeneID:80539275	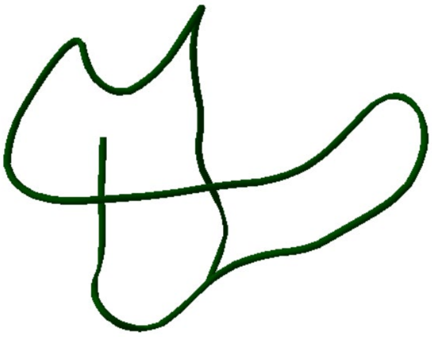
GeneID:80539276	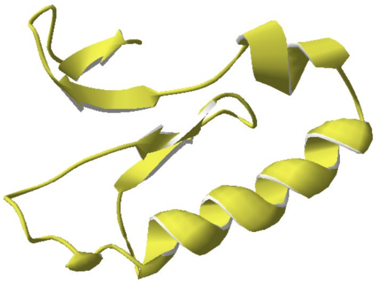
GeneID:80539277	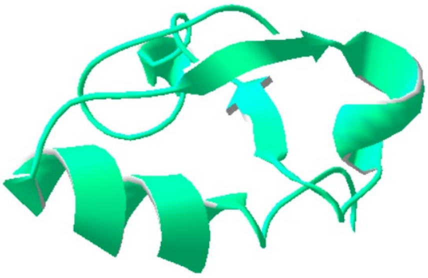
GeneID:80539278	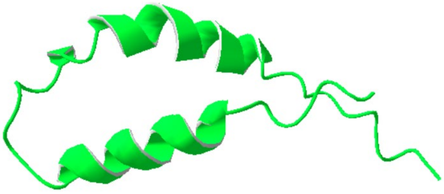

GENE_ID	3d structure
GeneID:80539279	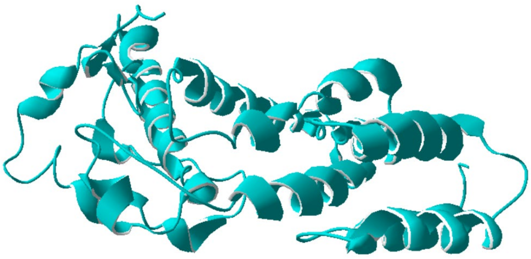
GeneID:80539280	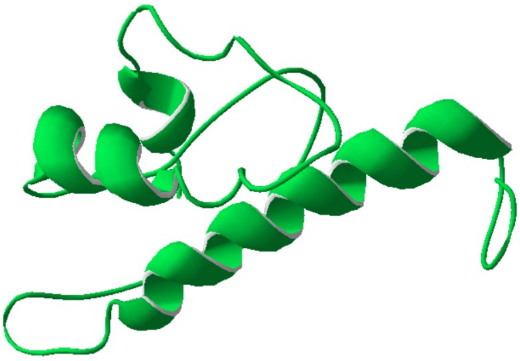
GeneID:80539281	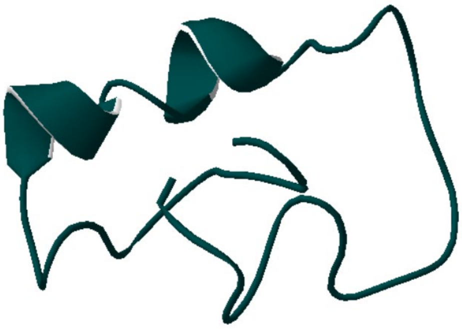
GeneID:80539282	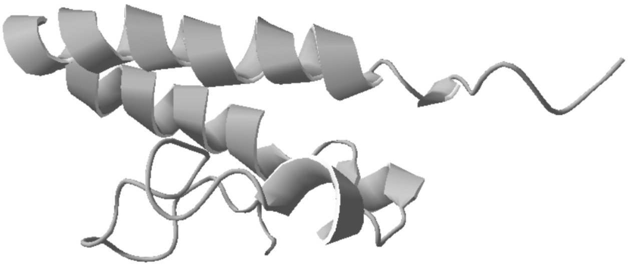
GeneID:80539283	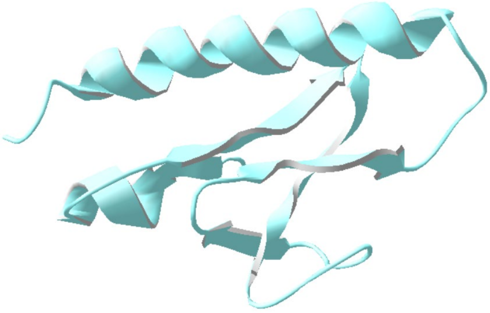

GENE_ID	3d structure
GeneID:80539284	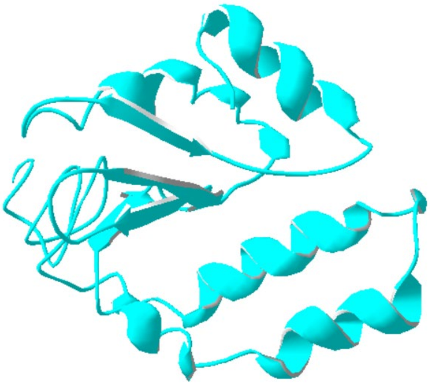
GeneID:80539285	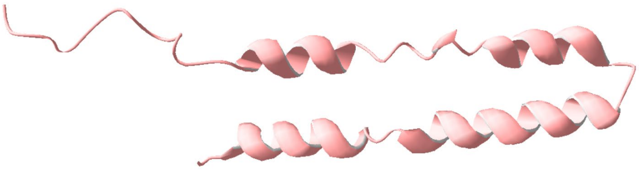
GeneID:80539257	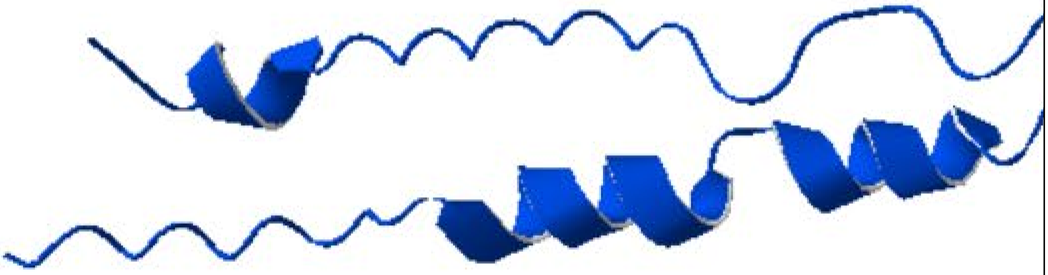

GENE_ID	3d structure
GeneID:80539287	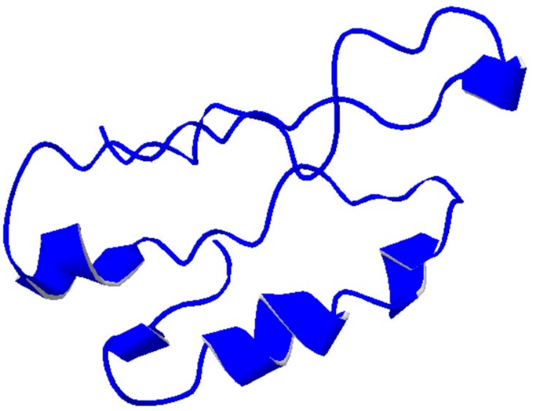
GeneID:80539288	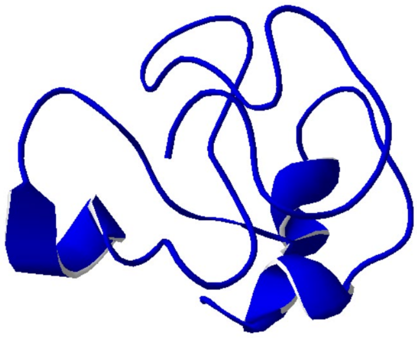
GeneID:80539289	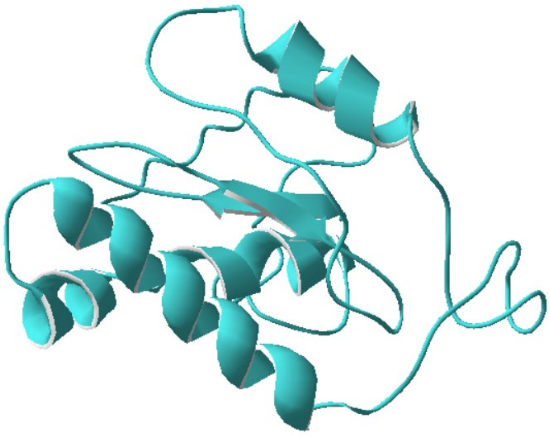
GeneID:80539290	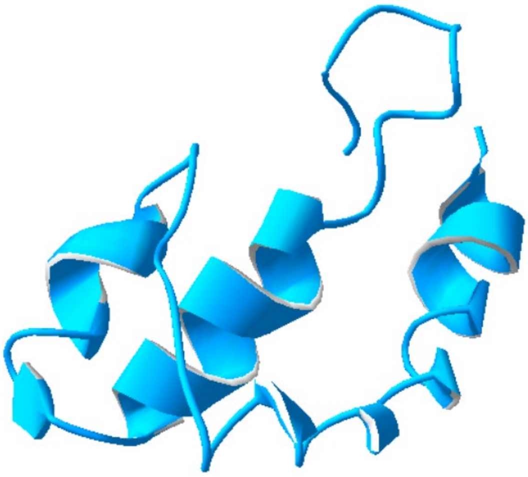

GENE_ID	3d structure
GeneID:80539291	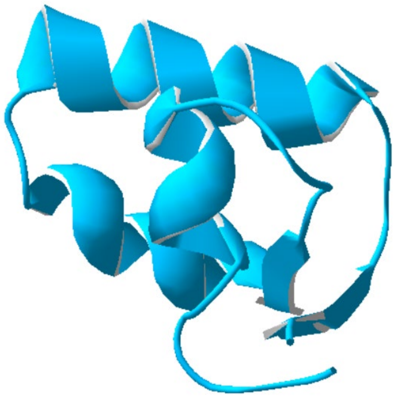
GeneID:80539292	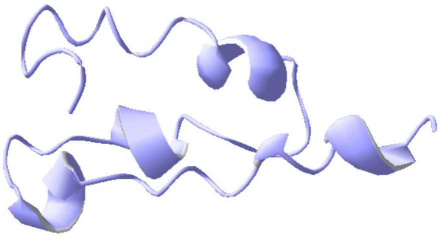
GeneID:80539293	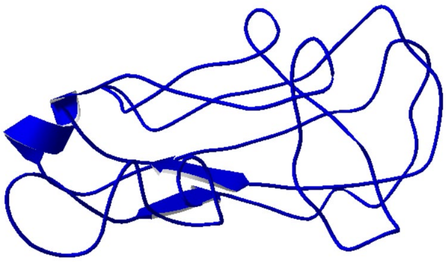
GeneID:80539294	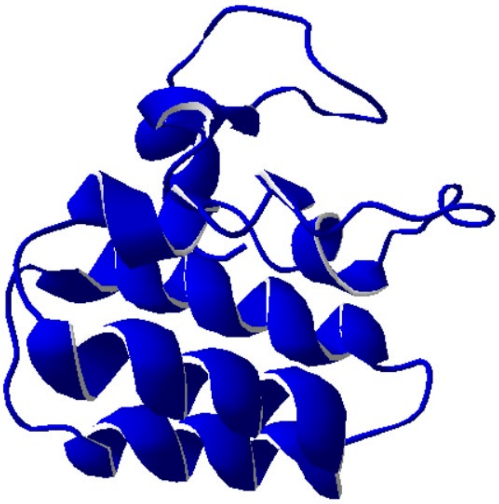

GENE_ID	3d structure
GeneID:80539295	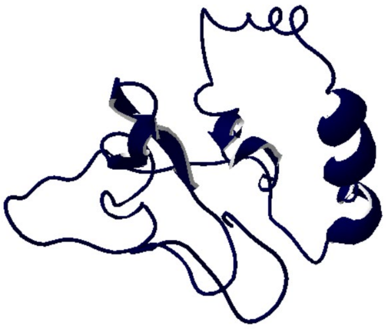
GeneID:80539296	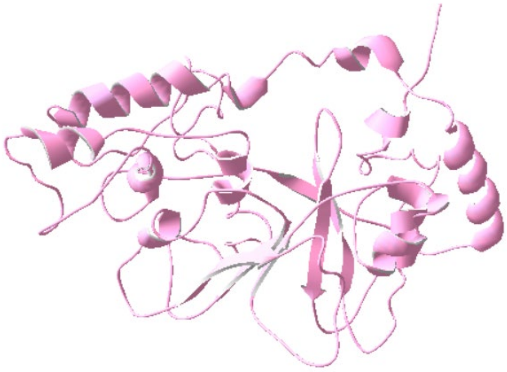
GeneID:80539297	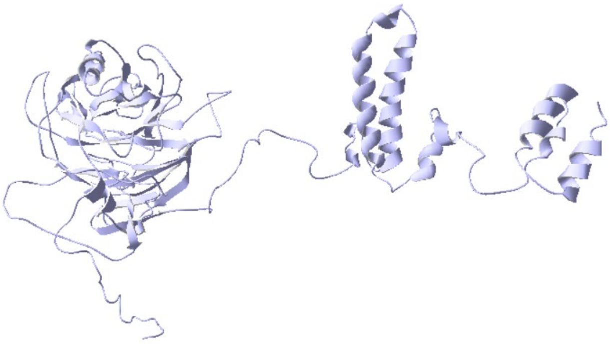
GeneID:80539298	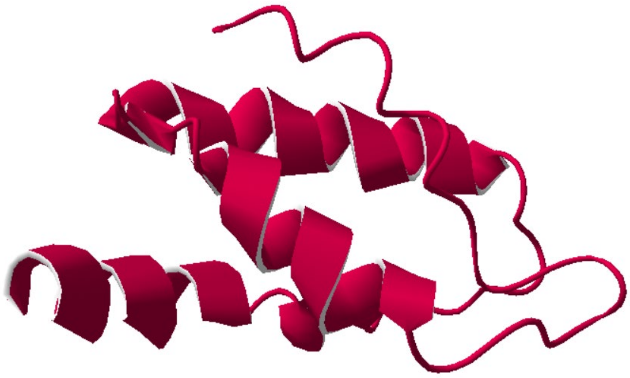
GeneID:80539299	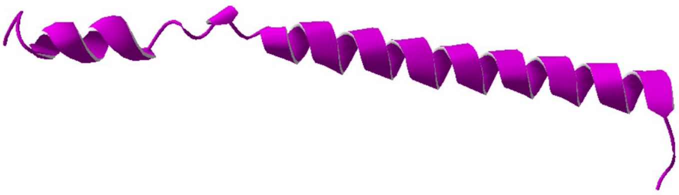

GENE_ID	3d structure
GeneID:80539300	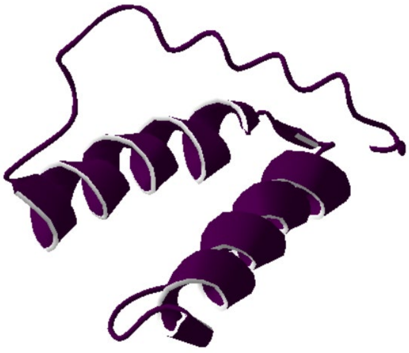
GeneID:80539301	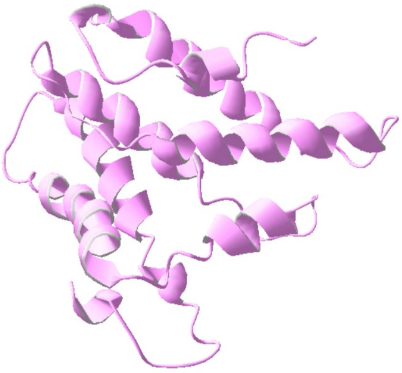
GeneID:80539302	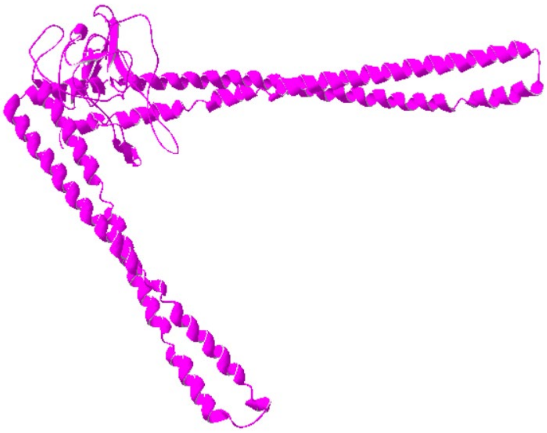

GENE_ID	3d structure
GeneID:80539303	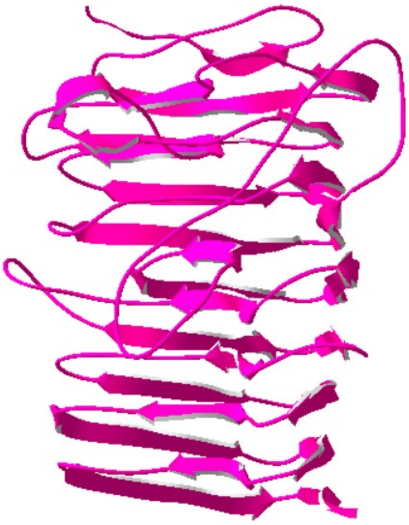
GeneID:80539304	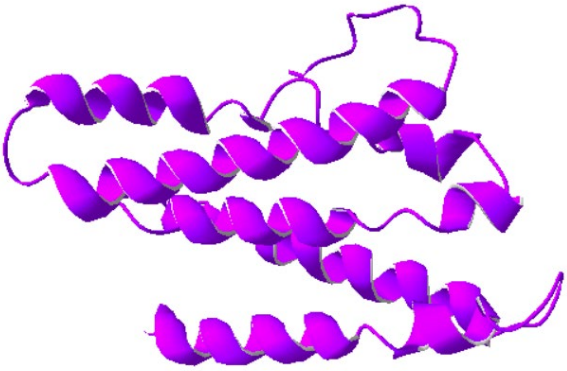
GeneID:80539305	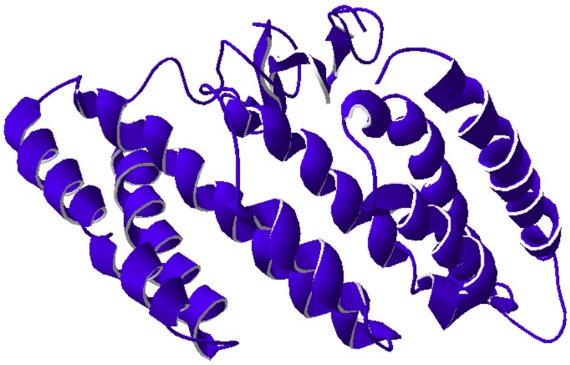

GENE_ID	3d structure
GeneID:80539306	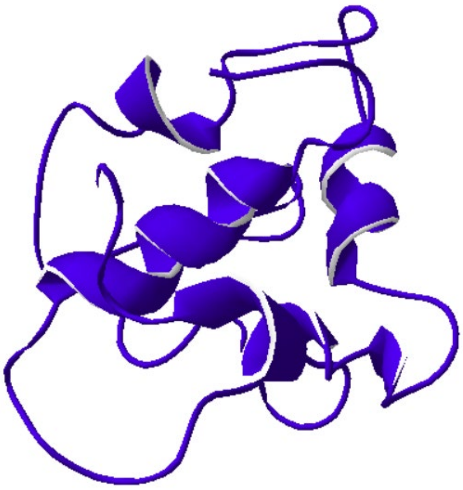
GeneID:80539307	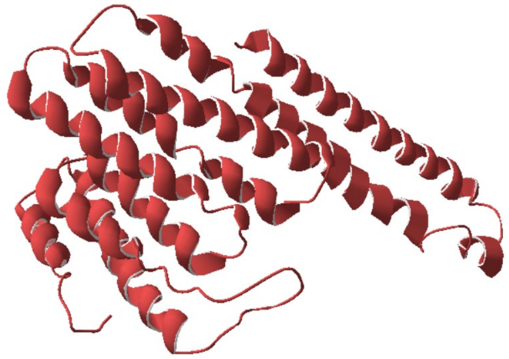
GeneID:80539308	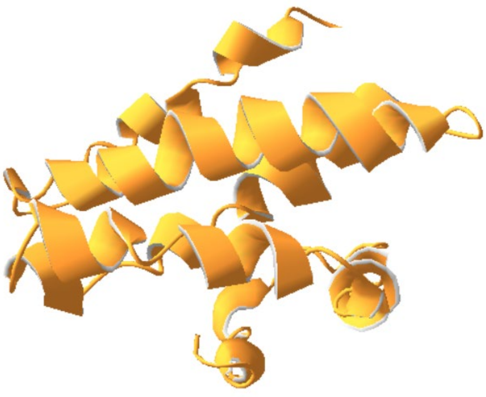
GeneID:80539309	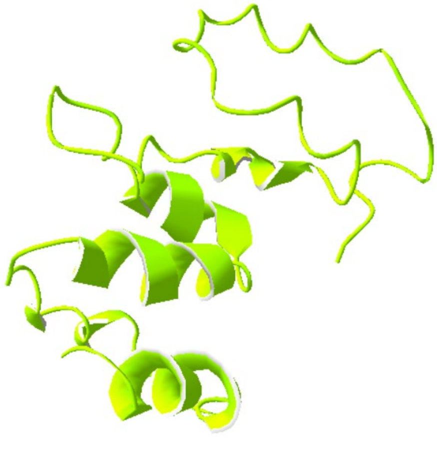

GENE_ID	3d structure
GeneID:80539310	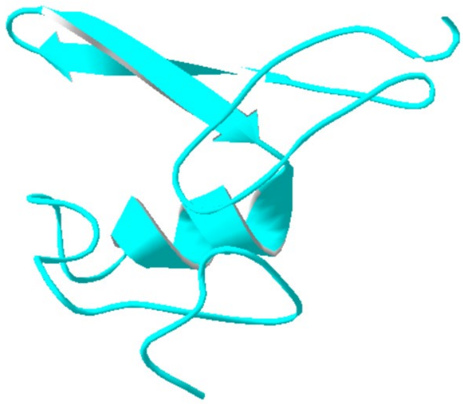
GeneID:80539311	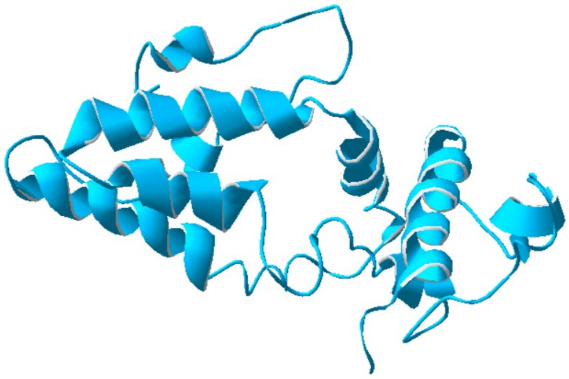
GeneID:80539312	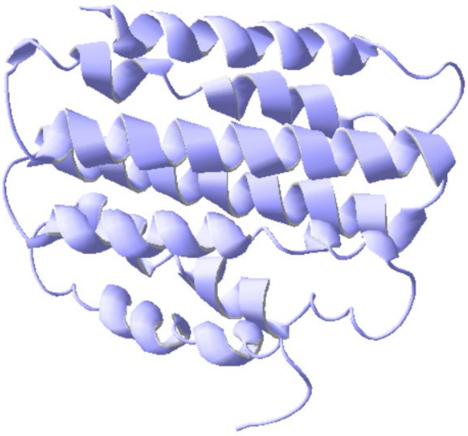
GeneID:80539313	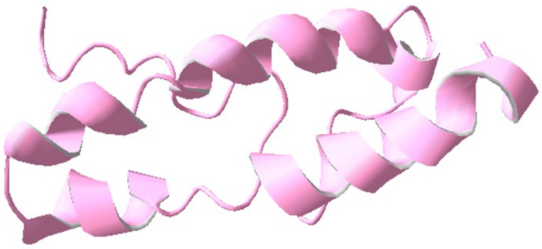

GENE_ID	3d structure
GeneID:80539314	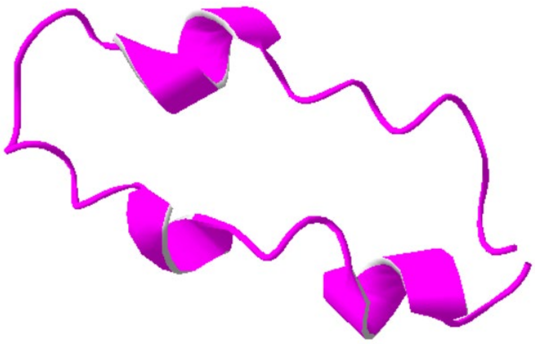
GeneID:80539315	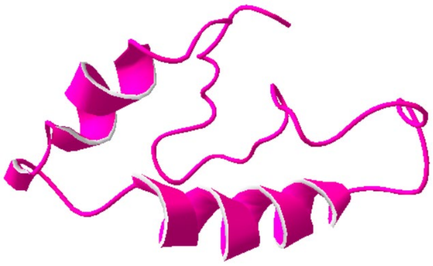
GeneID:80539316	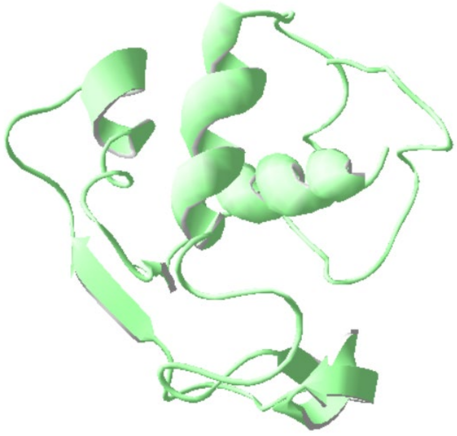
GeneID:80539317	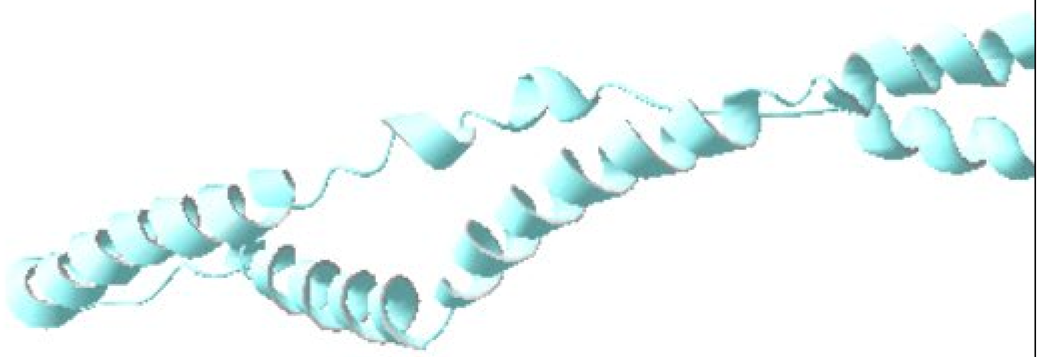

GENE_ID	3d structure
GeneID:80539318	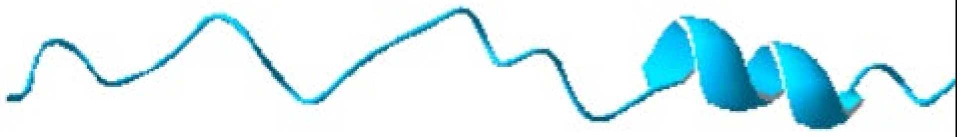
GeneID:80539319	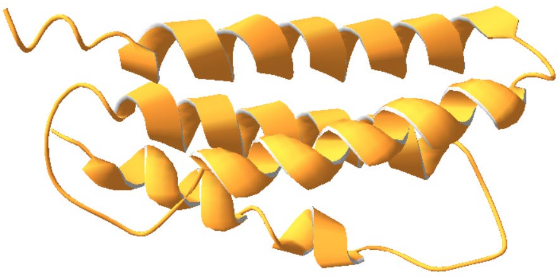
GeneID:80539320	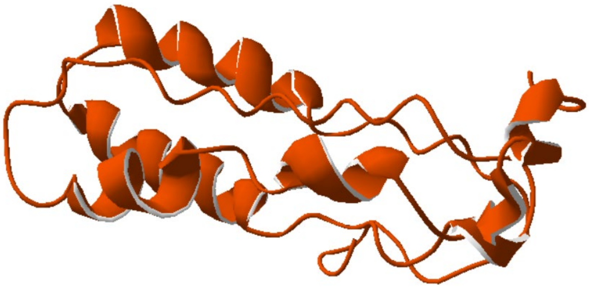
GeneID:80539321	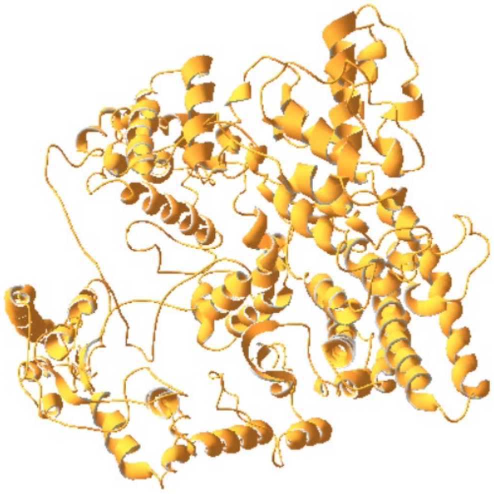

GENE_ID	3d structure
GeneID:80539322	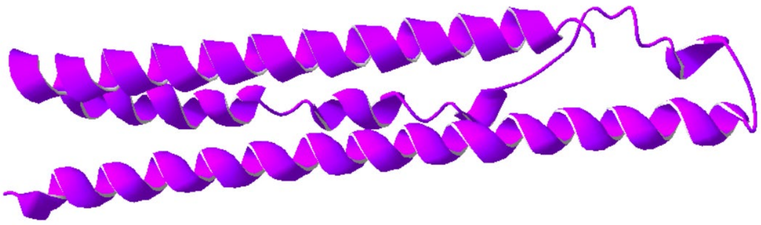
GeneID:80539323	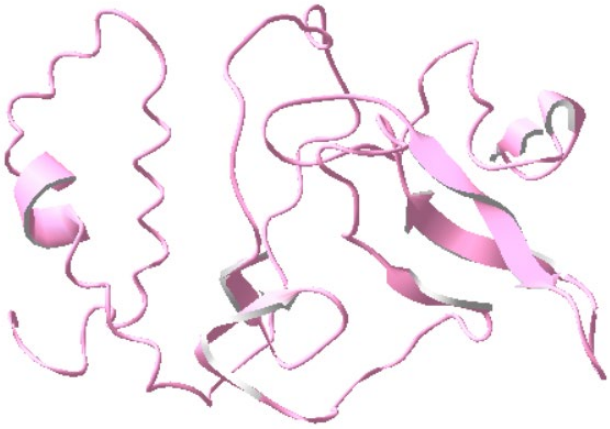
GeneID:80539324	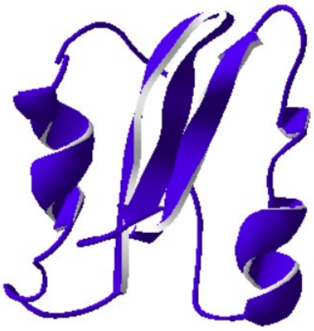

GENE_ID	3d structure
GeneID:80539325	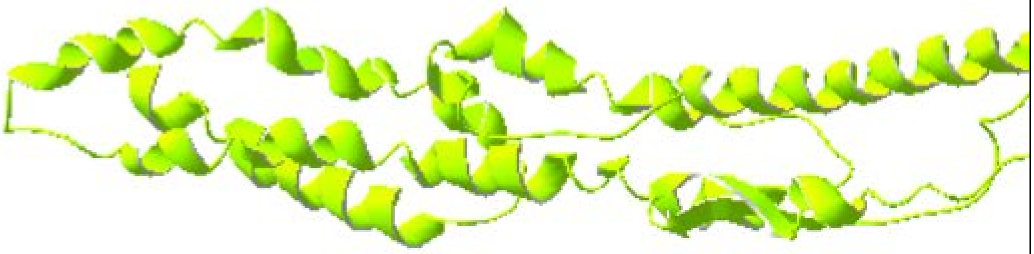
GeneID:80539326	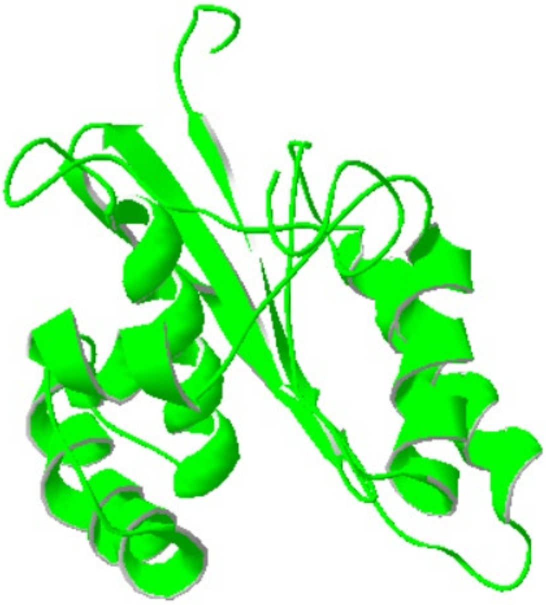

GENE_ID	3d structure
GeneID:80539327	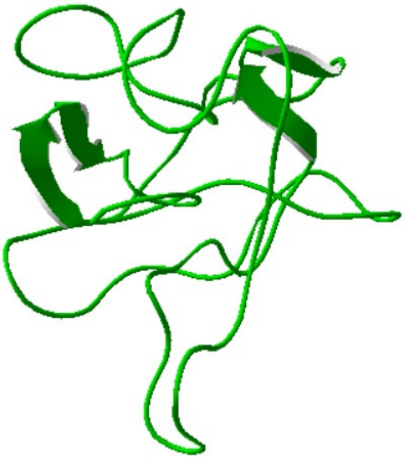
GeneID:80539328	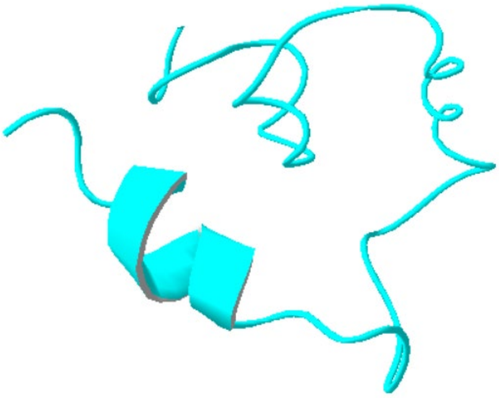
GeneID:80539329	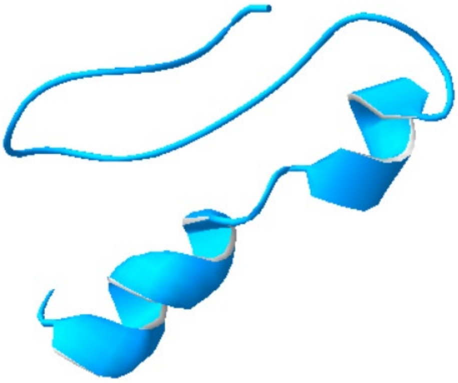

GENE_ID	3d structure
GeneID:80539330	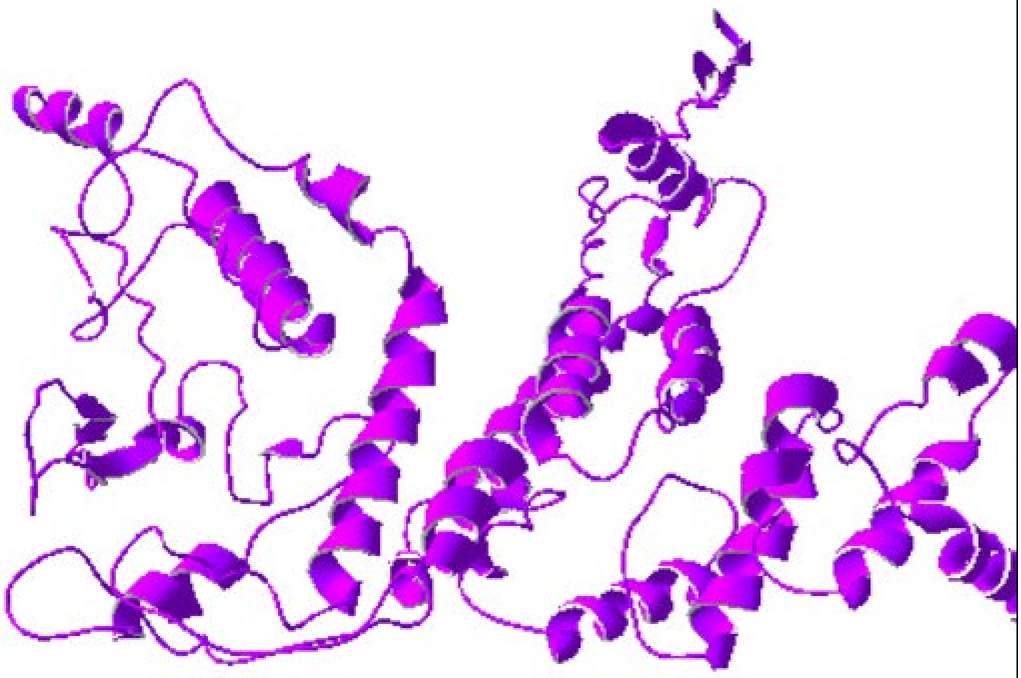
