# Supplementary material for: Yaravirus brasiliense genomic structure analysis and its possible influence on the metabolism
Source: Genet Mol Biol. 2025 Feb 7;48(1):e20240139. doi: 10.1590/1678-4685-GMB-2024-0139 (PMC11803573; doi:10.1590/1678-4685-GMB-2024-0139)
Supplement: Figure S2 - [file 1415-4757-GMB-48-1-e20240139-s6.pdf]

Genetics and Molecular Biology
DOI: https://doi.org/10.1590/1678-4685-GMB-2024-0139

Supplementary Material to "*Yaravirus brasiliense* genomic structure analysis and its possible influence on the metabolism"

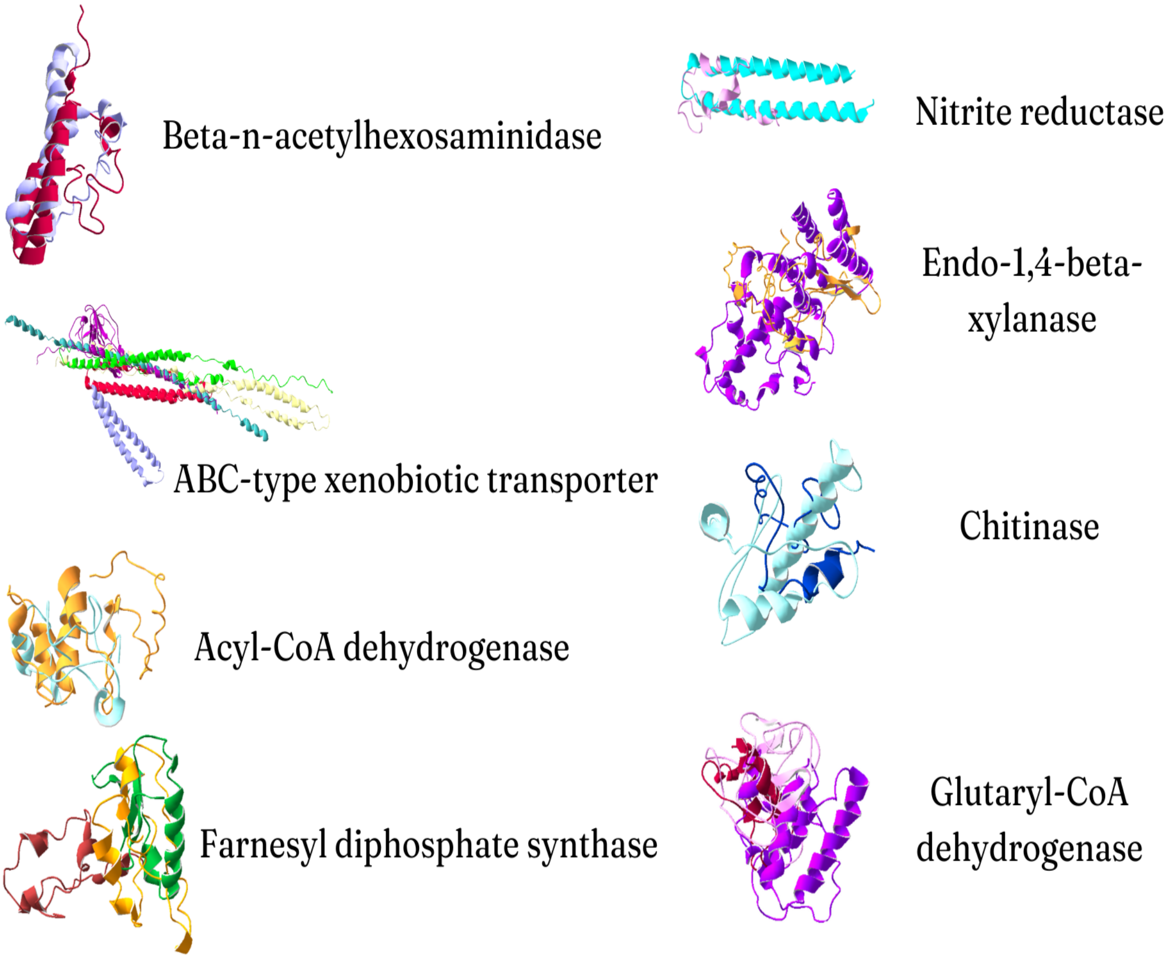

Figures S2 - All assembled proteins and their respective functions. Proteins related to nitrogen metabolism, mostly. Different colors mean different peptides. Not the same colors used in Table S4.
